# *De novo* identification of bacterial antigens of a clinical isolate by combining use of proteosurfaceomics, secretomics, and BacScan technologies

**DOI:** 10.3389/fimmu.2023.1274027

**Published:** 2023-11-30

**Authors:** Jinyue Yang, Xueting Zhang, Junhua Dong, Qian Zhang, Erchao Sun, Cen Chen, Zhuangxia Miao, Yifei Zheng, Nan Zhang, Pan Tao

**Affiliations:** ^1^ State Key Laboratory of Agricultural Microbiology, College of Veterinary Medicine, Huazhong Agricultural University, Wuhan, Hubei, China; ^2^ Key Laboratory of Prevention & Control for African Swine Fever and Other Major Pig Diseases, Ministry of Agriculture and Rural Affairs, Cooperative Innovation Center for Sustainable Pig Production, Huazhong Agricultural University, Wuhan, Hubei, China; ^3^ Hubei Hongshan Lab, Wuhan, Hubei, China; ^4^ Veterinary Diagnostic Laboratory, Neixiang Center for Animal Disease Control and Prevention, Nanyang, Henan, China; ^5^ Neixiang Animal Health Supervision, Neixiang Animal Husbandry Bureau, Nanyang, Henan, China

**Keywords:** emerging bacterial pathogens, highly immunogenic proteins, *de novo* identification, vaccine, *A. pleuropneumoniae*

## Abstract

**Background:**

Emerging infectious diseases pose a significant threat to both human and animal populations. Rapid *de novo* identification of protective antigens from a clinical isolate and development of an antigen-matched vaccine is a golden strategy to prevent the spread of emerging novel pathogens.

**Methods:**

Here, we focused on *Actinobacillus pleuropneumoniae*, which poses a serious threat to the pig industry, and developed a general workflow by integrating proteosurfaceomics, secretomics, and BacScan technologies for the rapid *de novo* identification of bacterial protective proteins from a clinical isolate.

**Results:**

As a proof of concept, we identified 3 novel protective proteins of *A. pleuropneumoniae*. Using the protective protein HBS1_14 and toxin proteins, we have developed a promising multivalent subunit vaccine against *A. pleuropneumoniae*.

**Discussion:**

We believe that our strategy can be applied to any bacterial pathogen and has the potential to significantly accelerate the development of antigen-matched vaccines to prevent the spread of an emerging novel bacterial pathogen.

## Introduction

Bacterial infectious diseases pose a serious threat to public health and cause huge economic losses worldwide (1, 2). While vaccines are widely recognized as the most effective countermeasure to prevent bacterial infections (3, 4), developing effective bacterial vaccines remains a significant challenge due to the presence of multiple serotypes and complex genomes. As demonstrated by methicillin-resistant *Staphylococcus aureus*, emerging bacterial epidemics pose a significant threat to human and animal populations (5). Therefore, there is an urgent need for standard methods to rapidly develop antigen-matched vaccines to prevent the spread of emerging novel bacteria or new serotypes. Although the traditional inactivated whole-pathogen vaccines may be effective against homologous strains, they are often associated with local or systemic side effects (6). In contrast, subunit vaccines, which consist of antigenic components of the pathogen rather than the entire pathogen (3), are much safer and represent the next-generation of vaccines.

The selection of protective antigens that can efficiently elicit immune responses is critical for the development of effective subunit vaccines. Bacterial capsular polysaccharides are major antigens of bacteria, but they are T cell-independent antigens that activate B cells directly without the help of T cells (7). As a result, the immune responses they induced are limited to the low affinity IgM antibodies (8) and may not provide long-lasting immunity or memory response. In contrast, bacterial immunogenic proteins are T-dependent antigens that require the help of T cells to activate B cells and might induce the durable immune responses (7, 9). The surface-exposed and secreted proteins of bacteria, such as adhesins, invasions, and immunomodulators (10), play crucial roles in their infection process. These proteins are exposed to and are readily recognized by host’s immune system (10), making them ideal targets for the development of bacterial vaccines.

Many techniques have been developed to identify bacterial antigenic proteins, such as reverse vaccinology (RV) and proteomic techniques. RV uses bioinformatics to analyze the entire genome sequence for the genes that are likely to encode surface-exposed or secreted proteins (11). These proteins are then expressed and screened, individually, for their ability to induce immune protection in animal models (11). RV was first applied to *Neisseria meningitidis*, and 350 potential antigens were identified and their protective efficacy were evaluated in animal models (11). Eventually, three of the seven identified protective antigens were used to develop the Bexsero vaccine (11, 12), which was approved by the US Food and Drug Administration (FDA) in 2015 (13), demonstrating the potential of reverse vaccinology. However, identifying seven protective antigens from 350 candidates is a time-consuming and laborious process (11). Alternatively, bacterial surface proteins can be identified by proteomic approaches such as the surface shaving technique, which uses proteases to partially degrade the surface proteins of intact bacteria (14). The generated peptide fragments were released from bacteria and identified by mass spectrometers. The surface proteins can also be labeled with membrane-impermeable biotin, enriched by avidin after bacterial cell lysis, and then analyzed via mass spectrometry (15). While these proteomic methods are attractive for identifying bacterial surface proteins, it should be noted that Gram-negative bacteria have thin cell walls and are prone to lysis (16), which can result in identifying false-positive cytoplasmic proteins.

Recently, we developed BacScan, a genome-wide technology for the identification of bacterial highly immunogenic proteins (HIPs), as demonstrated using *Streptococcus suis* (17). The core genes of *S. suis* were split into 600-bp equal-length DNA fragments with a 300-bp overlap between adjacent fragments and inserted into T7 phage genome to generated a T7 display library. T7 phages displaying *S. suis* antigenic protein were immunoprecipitated by incubation with sera and protein A/G beads. The immunogenic proteins were subsequently identified by high-throughput sequencing of the enriched T7 phages (17). BacScan can quickly identify immunogenic proteins that are conserved among different serotypes using bacterial core genome, which was defined by comparing the genomic sequences of hundreds of strains of the same bacterial species. However, the core genome is not always available for the less-studied bacteria, especially emerging bacteria isolated in clinical settings.

**Figure 1:**
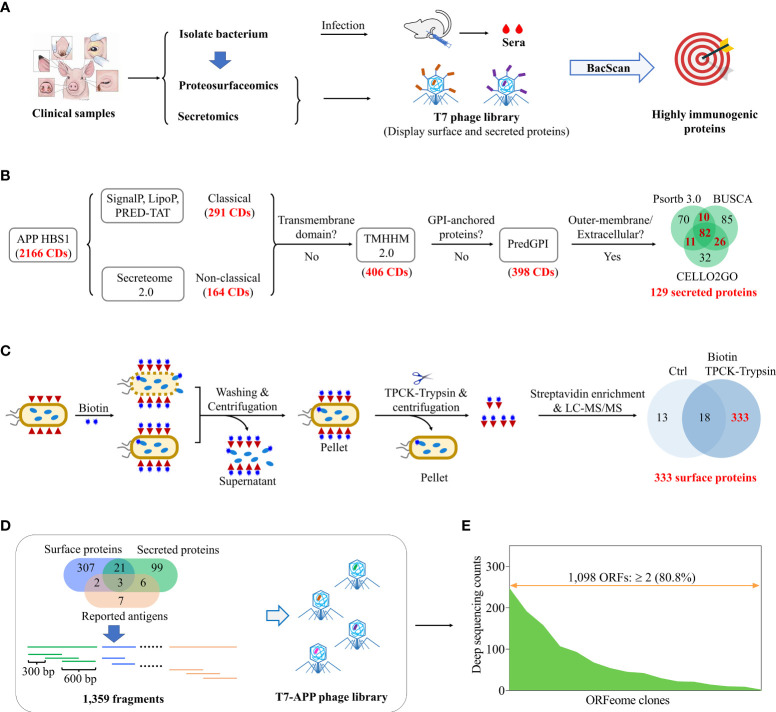
Construction of a T7 library displaying surface and secreted proteins of *A*. *pleuropneumoniae* for *de novo* identification of immunogenic proteins. **(A)** Schematic diagram shows identification of highly immunogenic proteins by combing use of proteosurfaceomics, secretomics, and BacScan technologies. Pathogenic bacteria were isolated from clinical samples and used to infection animals to prepare strain-specific sera. The bacterial surface proteins and secreted proteins were identified by proteosurfaceomics and secretomics analysis, respectively. A T7 phage library displaying surface proteins and secreted proteins was constructed and used to screen highly immunogenic proteins using BacScan. **(B)** Identification of *A*. *pleuropneumoniae* secreted proteins using bioinformatics. Proteins were sequentially examined for the secreted signal peptide, transmembrane domain, GPI-anchored, and subcellar location. **(C)** Identification of *A*. *pleuropneumoniae* surface proteins by *in situ* biotinylation labeling, TPCK-trypsin digestion, streptavidin-agarose pull-down, and mass spectrometry analysis. Unlabeled *A*. *pleuropneumoniae* cells treated with TPCK-trypsin digestion followed by streptavidin pull-down were used as a control for mass spectrometry. **(D)** Schematic diagram showing the construction of a T7-*A. pleuropneumoniae* (APP) phage library. The genes encoding surface and secreted proteins were divided into 600-bp DNA fragments with a 300-bp overlap between adjacent fragments and inserted into T7 phage genome to generated a T7 display library. **(E)** Illumina sequencing of T7-APP phage library.

Here, we aimed to develop a universal pipeline to identify the immunogenic proteins *de novo* from a clinically isolated novel *Actinobacillus pleuropneumoniae* strain by combining of proteosurfaceomics, secretomics, and BacScan technologies (**Figure 1A**). *A. pleuropneumoniae* is a highly contagious gram-negative bacterium and causes severe respiratory disease in the pig (18). At least 19 A*. pleuropneumoniae* serotypes have been reported (19), however, there are only 36 complete genome sequences available in the NCBI database as of May 2023. The proteosurfaceomics and secretomics are used to identify bacterial surface-exposed proteins and secreted proteins respectively, which are used to construct a T7 display library for BacScan screening (**Figure 1A**). For a proof concept, we isolated a highly virulent *A. pleuropneumoniae* HBS1 from a diseased pig. The surface-exposed proteins of HBS1 were *in situ* biotinylated, released by partial digested with TPCK-treated trypsin, and identified by mass spectrometry analysis after streptavidin-agarose pulldown (**Figure 1C**). The potential secreted proteins were identified as showed in **Figure 1B**. The T7 display library containing surface proteins and secreted proteins were then generated and used for BacScan screen against *A. pleuropneumoniae*-specific sera. We identified 15 highly immunogenic proteins, and three of them provided immunized mice partial protection against *A. pleuropneumoniae* challenge. In particular, the HBS1_14, which is highly conserved among all serotypes, is a promising antigen for development of a universal *A. pleuropneumoniae* subunit vaccine. Our study establishes a rapid and high-throughput universal pipeline for identifying bacterial immunogenic proteins *de novo* from a clinical isolate and can be applied broadly to any other bacterial pathogens.

## Materials and methods

### Bacterial strains and kits

*Escherichia coli* DH5α chemically competent cells were used for the plasmid construction. *E. coli* BL21 (DE3) RIPL cells were utilized for protein expression. The T7Select Packaging kit (Merck, USA) was used for the construction of phage library. A commercial ApxIV-ELISA kit (Keqian Biotech, China) was used to detect *A. pleuropneumoniae* in porcine serum samples.

### Serum samples

A total of 21 swine serum samples were collected from pig farms. *G. parasuis* positive pig sera (20) were generously provided by Dr. Xiaojuan Xu of Huazhong Agricultural University. *S. suis*-positive porcine sera (n=5) and piglet sera negative for common swine pathogens (n=5) were prepared in our previous study (17). *A. pleuropneumoniae*-specific sera were prepared with specific pathogen-free mice as described below. 20 six- to eight-week-old female BALB/c mice obtained from the Laboratory Animal Center of Huazhong Agricultural University were randomly divided into four groups, with 5 mice per group. Mice in group 1 were intraperitoneally immunized with inactivated APP HBS1 with aluminum adjuvant at weeks 0, 2, and 3. Mice in groups 2 and 3 were infected with 100 μL of PBS containing 2.5×10^6^ CFU of *A. pleuropneumoniae* HBS1 by i.p. and i.n. routes, respectively. Mice in group 4 were inoculated with an equivalent volume of PBS and used as negative controls. Serum samples were collected on days 14, 21, and 28.

### Isolation and characterization of *A. pleuropneumoniae* HBS1

Six lung tissues collected from pigs suspected to have pleuropneumonia were used to isolate *A. pleuropneumoniae* using Tryptic Soy Agar (TSA) (Difco, USA) supplemented with 5% Fetal Bovine Serum (FBS) (Sijiqing, China) and 10 μg/mL nicotinamide adenine dinucleotide (NAD) (Biosharp, China). After 24 hours incubation at 37°C, colonies were test individually with *A. pleuropneumoniae*-specific primers (**Table S2**) (21). One *A. pleuropneumoniae* colony from each sample were picked and further purified. The serotype was determined using serotype-specific primers (**Table S2**) (22–24). One strain (HBS1) belongs to the highly pathogenic serotype 1 was selected for the following studies. To determine the 50% lethal dose (LD_50_) of HBS1 in mice, freshly harvested cultures were washed and suspended in PBS. Mice in groups 1 to 4 (5 mice per group) were intraperitoneally infected with 100 μL of HBS1 at various doses ranging from 3.75×10^6^ to 3×10^7^ CFU and monitored for mobility and mortality for 7 days. The control mice were injected with an equivalent volume of PBS (**Table S1**). The LD_50_ was calculated with the method of Reed and Muench (25).

To determine the pathogenicity of the HBS1, six- to eight-week-old mice (n=3) were intraperitoneally injected with a lethal dose of HBS1 (3×10^7^ CFU). Mice (n=3) injected with an equivalent volume of PBS were used as controls. Mice were euthanized 12 hours after infection to determine the tissue bacterial load and pathological changes. Mouse hearts, livers, spleens, lungs, and kidneys were collected and divided into two portions. One portion was used to determine tissue bacterial load and the second portion was used for pathological changes as described below.

### Whole-genome sequencing

Genomic DNA was extracted from *A. pleuropneumoniae* HBS1 using the E.Z.N.A.^®^ Bacterial DNA kit (OMEGA, Bio-Tek), and 0.5 μg DNA was used for library preparation and sequencing. Quality control of the sequencing data was checked using Sickle to remove low-quality and short reads. The filtered high-accuracy Illumina data reads (Q30>85%) were assembled using Unicycler (26) to obtain high-quality bacterial genome scaffolds (contigs), which were used to assemble the complete genome graphs using Nanopore data. Finally, the assembled genomes were further polished using Pilon with the Illumina data to obtain the final genomes with higher accuracy. The genome sequence was submitted to NCBI database under the GenBank accession number CP115971.

### Surface shaving of live cells

The surface proteins were identified by enzymatic shaving as previously described (27). Briefly, the APP HBS1 strain was cultured in 10 mL of TSB at 37°C until reaching an optical density of 0.4. The bacteria were harvested by centrifugation at 3,500×g for 10 minutes at 4°C, followed by six washes with ice-cold PBS (pH 7.4). The cell pellets were resuspended in 1 mL of PBS supplemented with 30% sucrose and 10 μg/mL of TPCK-trypsin (Promega, USA). Proteolytic reactions were conducted at 37°C for 10, 20, 30, 40, or 50 minutes and terminated with 0.1% formic acid. The mixtures were centrifuged at 3,500×g for 10 minutes at 4°C. The supernatants were further centrifuged at 34,000×g for 30 minutes at 4°C and filtered through a 0.22 μm filter. Peptide fractions were concentrated using a vacuum concentrator (Eppendorf, USA) for mass spectrometry analysis. Negative control cells were treated with the same procedure but without TPCK-trypsin.

### Enrichment of the surface proteins by biotinylation and streptavidin-agarose pulldown

The surface proteins were labeled with Sulfo-NHS-LC-Biotin following the manufacturer’s instructions (Pierce). Briefly, freshly harvested bacterial cells were washed and resuspended in 1 mL of PBS (pH 8.0) containing 2 mM Sulfo-NHS-LC-Biotin. Biotinylation reactions were performed on ice for 10, 20, 30, 40, 50, or 60 minutes, and the excess biotin was quenched with PBS containing 500 mM glycine. The cell pellets were resuspended in ice-cold PBS containing 1% NP-40 and sonicated on ice for 2 minutes (300 W; pulse on, 10 s; and pulse off, 15 s) using an ultrasonic processor (Sonics & Materials, Newtown, CT). The supernatants were filtered through a 0.22 μm filter after centrifugation at 34,000×g for 30 minutes to remove cell debris (28). The biotinylated proteins were incubated with the streptavidin agarose for 60 minutes with gentle shaking on ice. After six washes with PBS/NP-40, the biotinylated proteins were eluted by adding 50 µL of SDS sample buffer. The control was treated with the same procedure but without Sulfo-NHS-LC-Biotin.

### Enrichment of surface-exposed proteins by combing use of *in situ* biotinylation, TPCK-trypsin digestion, and streptavidin-agarose pulldown

The surface-exposed proteins were *in situ* biotinylated as described above. After six washes, the biotinylated cells were resuspended in 1 mL of PBS (pH 7.4) containing 30% sucrose and 10 μg/mL TPCK-trypsin and incubate at 37°C for 20 minutes to release the biotinylated surface-exposed proteins. After terminating the proteolytic reaction, the supernatant was collected after centrifugation as described above. The biotinylated peptides were purified using streptavidin-agarose beads, as detailed in above.

### Western blot

The biotinylated protein samples were separated on a 12% SDS-PAGE and transferred onto PVDF membranes (Millipore, Boston, MA). After blocking with 5% bovine serum albumin (BSA), the membranes were incubated with HRP-conjugated streptavidin (Abbkine, China). After three washes with Tris-buffered saline containing 1% tween-20 (TBS-T), the protein signals were visualized using enhanced chemiluminescence (ECL) (Bio-rad, USA).

### Liquid chromatography-tandem-mass spectrometry

Protein samples were analyzed by liquid chromatography-tandem-mass spectrometry (LC-MS/MS) as previously described (28). Briefly, proteins were separated on an SDS-PAGE gel, and the gel slices were distained, dehydrated, and then subjected to in-gel digestion with trypsin. The resulting peptides were separated and analyzed using a Q ExactiveTM series mass spectrometer (Thermo Fisher) with Nanospray Flex™ (ESI). The acquired spectra were searched against the Uniprot database using the search engines: Proteome Discoverer 2.2 (PD 2.2, Thermo). The protein identification lists were filtered to maintain a peptide false discovery rate (FDR) less than 1% for ensuring high accuracy. The raw data, and database search results were deposited in ProteomeXchange (http://www.proteomexchange.org/) under project accession number PXD046029.

### Prediction secreted proteins

Classical secretory pathways were predicted by SignalP 5.0, LipoP 1.0, and PRED-TAT to analyze the general secretion (Sec) pathway and the twin-arginine translocation (Tat) system (29). Proteins identified by at least two of the three methods were classified as classically secreted proteins. Non-classical secretory pathways were analyzed using SecretomeP 2.0 (30). Transmembrane helices were predicted using TMHMM 2.0 (31), and proteins lacking transmembrane helices were analyzed using PredGPI (32). Non-GPI anchored proteins were further analyzed for their subcellular localization using PSortb 3.0 (33), CELLO2GO (34), and BUSCA (35). A protein is considered to be a secreted protein if it is located in the outer membrane or extracellular space as determined by at least two of the three methods.

### Construction of T7-APP phage library

The encoding regions of surface-exposed and secreted proteins were PCR amplified from the APP HBS1 genomic DNA (All primers are available from the corresponding author on request). Each gene was divided into 600-nucleotides (nt) fragments with a 300-nt overlap between adjacent fragments. Fragments containing *EcoR I/Hind III* or *BamH I/Not I* were modified by introducing synonymous mutations to disrupt the restriction sites of these restriction endonucleases. The PCR products were pooled in equal amounts, digested with *EcoR I/Hind III* or *BamH I/Not I*, and cloned into T7Select 10-3b vector linearized with the corresponding restriction endonucleases. The ligation products were packaged into infectious T7 phage particles using the T7Select^®^ Packaging Kit (Novagen), following the manufacturer’s protocol. The quality of phage library was confirmed by next-generation sequencing.

### Phage immunoprecipitation and sequencing

Phage immunoprecipitation and sequencing were performed as previously described (17). Briefly, 1.5-mL Eppendorf tubes were blocked with 3% BSA in TBS-T overnight at 4°C. Two μg of serum IgG and 1 mL of 2×10^8^ PFU T7 phages were added to each tube and incubated on a rotator at 4°C for 18 hours to facilitate antibody-phage binding. Subsequently, 40 μL of Protein A/G magnetic beads (Pierce) were added to each tube and incubated on a rotator for 4 hours at 4°C. After three washes with washing buffer (50 mM Tris-HCl, pH 7.5, 150 mM NaCl, 0.1% NP-40), The beads were resuspended in 40 μL of pure water. The phages were lysed by heating at 95°C for 10 minutes and frozen at −80°C for further analysis.

The DNA multiplexed Illumina sequencing protocol was conducted as previously described (17). Briefly, the *A. pleuropneumoniae* gene fragments were amplified by two rounds of PCR using the lysed phage as a template. The first round of PCR used PCR1-F and PCR 1-R primers (**Table S3**). The PCR products were used as templates for the second round of PCR with a forward primer (PCR2-F) and a unique indexing primer (**Table S3**). The PCR products were sequenced at the National Key Laboratory of Crop Genetic Improvement, Huazhong Agricultural University. The generalized Poisson model was used to normalize the data and remove background bias. The Illumina sequencing data was deposited in Sequence Read Archive (SRA) of the NCBI database with the BioProject accession number PRJNA1026755.

### Plasmid construction and protein purification

HBS1_01 and HBS1_07 were cloned into expression plasmid pET22b, whereas the remaining 13 HIPs, ApxI, and ApxII were individually cloned into expression plasmid pET32a using either restriction ligation (Thermo, USA) or Gibson assembly (Vazyme, China) methods. The primers used for cloning were listed in **Table S4**. All the plasmids were sequenced for accuracy before transformed into *E. coli* BL21 (DE3) RIPL. The recombinant HIPs were induced with 1mM isopropyl-β-D-thiogalactoside (IPTG) at 30°C for 3-4 hours when the OD_600_ of culture reached 0.8. and purified as previously described (36). Briefly*, E. coli* cells were collected and resuspended with binding buffer (20 mM Tris-HCl pH 8.0, 100 mM NaCl, 10 mM imidazole, and 5 μg/mL DNase I). After cell lysis using a high-pressure cell disruptor, cell debris was removed by high-speed centrifugation. The recombinant HIPs were purified using a HisTrap column. The purity of the eluted HIPs was analyzed by SDS-PAGE, and protein concentrations were quantified using the Bradford Protein Assay Kit (Beyotime, China).

### Enzyme-linked immunosorbent assay

Antigen-specific antibodies in serum samples were determined using an enzyme-linked immunosorbent assay (ELISA). Briefly, 96-well ELISA plates were coated with proteins (200 ng/well) and blocked with 3% BSA in PBS containing 0.05% Tween-20 (PBS-T) for 1 hour at 37°C. 100μL of 2-fold serial dilution of serum were added to each well, and the plate was incubated at 37°C for 1 hour. HRP-conjugated goat anti-mouse antibodies specific for IgG (Abbkine, China), IgG1 (Abclonal, China), IgG2a (Abclonal, China), or HRP-conjugated goat anti-pig IgG (Solarbio, China) were then added to each well and incubated at 37°C for 1 hour. After five washes, tetramethylbenzidine (TMB) substrate was added and incubated for 10-15 minutes at room temperature in the dark. The colorimetric reaction was stopped with 2 M H_2_SO_4_, and the absorbance at 450 nm was measured using a microplate reader (BMG LABTECH, Germany).

### Homology analysis of HIPs

The whole genome sequences of the 18 different serotypes of *A. pleuropneumoniae* were downloaded from NCBI genome database to analyze the conservation of the fifteen HIPs in each serotype. A heatmap was used to visualize and present the results. The homology of HIPs was analyzed by BLAST search of NCBI. The search was restricted to exclude *Actinobacillus* (taxid: 713) but included *E. coli* (taxid: 562), *G. parasuis* (taxid: 738), *S. suis* (taxid: 1307), and *P. multocida* (taxid: 747). The search was conducted using the “blastp” tool with a non-redundant database, hit list size of 5000, and an e-value threshold of 10. A representative strain was identified from each bacterial species for homology analysis. The results were presented in the form of a heatmap. All identified HIPs were also BLASTed using the same parameters to exclude the HIPs with more than 30% sequence homology to pig proteins of (taxid: 9823) to exclude any potential cross-reactivity with swine proteins.

### Immunization and challenge

To determine the efficacy of 15 HIPs, 65 mice were randomly divided into 13 groups, with five mice in each group. Fifty μg of each recombinant HIP was mixed with 25μL of white oil adjuvant (Montanide ISA 201 VG) and injected intramuscularly (i.m.) into a mouse at weeks 0 and 2. Mice (n=5) administered with an equal volume of PBS were used as negative controls. Two weeks after the boost, mice were challenged intraperitoneally with a lethal dose of *A. pleuropneumoniae* HBS1 (2.5×10^7^ CFU) and monitored for mortality for seven days.

For the multivalent vaccine study, four groups of mice (n=5) were i.m. administered two times at weeks 0 and 2 with ApxI+ApxII, ApxI+ApxII+SRP (HBS1_14), Porcilis APP, and PBS, respectively. Two weeks after boost, mice were i.p. challenged with 1×10^8^ CFU *A. pleuropneumoniae* HBS1 and monitored for mortality for seven days. To further determine the efficacy of these three different vaccines by analyzing tissue bacterial load and histopathologic changes, 27 mice were immunized and challenged as described above. Three mice from each group were sacrificed at 12-, 36-, and 72-hours post challenge to determine the tissue bacterial load and to analyze the histopathologic changes as described below. Three mice administered with PBS and challenged with the same dose of *A. pleuropneumoniae* HBS1 were sacrificed at 12-hour post challenge as controls. Three naïve mice were used as blank controls.

### Tissue bacterial load and histopathologic analysis

The hearts, livers, spleens, lungs, and kidneys were collected from mice treated as described above. The samples were divided into two portions to determine the tissue bacterial load and histopathological changes, respectively. One portion was weighed, ground, mixed with an equal volume of PBS (1 mL PBS per 0.1 g tissue sample), and filtered. 100 μL of 10-fold serially diluted tissue solutions were plated on TSA agar plates supplemented with FBS and NAD. The plates were then incubated overnight at 37°C. The number of bacterial colonies on each plate was counted to calculate the number of bacterial per gram of tissue. The second portion of samples were fixed in 10% formalin, dehydrated through an ethanol series, embedded in paraffin wax, and cut into 4-μm-thick sections. The sections were deparaffinized, stained with hematoxylin-eosin, and observed under an optical microscope (Nikon, Japan) for histopathological changes.

### Statistical analysis

Statistical analysis was performed by Student t-test using Prism Graphpad software 8.0. Multi-group comparisons were assessed using one-way analysis of variance (ANOVA). Kaplan-Meier survival curves were analyzed by log-rank test (Mantel-Cox). Statistically significant differences were determined when P values were < 0.05.

## Results

### Construction of a T7 phage library displaying surface and secreted proteins of *A. pleuropneumoniae*

To develop a rapid method for identifying candidate protein antigens *de novo* from a clinically isolated new bacterial strain, we first isolated six strains of *A. pleuropneumoniae* from the lungs of pigs having pleuropneumonia. PCR serotyping indicated that one strain, named HBS1, belongs to the highly pathogenic serotype 1 and was therefore selected for the following studies (**Figures S1A, B**). The HBS1 is lethal to mice with the median lethal dose of 1.2×10^7^ colony-forming units (CFU) (**Table S1**). The tissue distribution of HBS1 and pathological damages of mice after infection were also determined (**Figures S1C–E**) (see **Supplementary** for the detail).

Bacterial surface-associated proteins and secreted proteins are exposed to the host immune system and tend to be more immunogenic (10). Therefore, we focus on these proteins and use them to construct a T7 phage display library for BacScan analysis (**Figure 1A**). To identify secreted proteins in silico, the whole genome of HBS1 was sequenced and deposited in the NCBI database under the GenBank accession number CP115971. Among all the 2,166 proteins encoded within HBS1 genome, 129 secreted proteins (**Figure 1B**, **Table S6**) of HBS1 were identified using a pipeline as described previously (29–35).

Surface-exposed proteins of *A. pleuropneumoniae* HBS1 were first identified using bacterial surface-shaving technique (14) (**Figure S2A**). HBS1 cells were digested with TPCK-treated trypsin for 20 min to release surface-exposed proteins (**Figure S2B**), which were subsequently identified using mass spectrometry. A total of 549 potential surface-exposed proteins were identified, however 394 (71.8%) proteins were also found in the untreated control group (**Figure S2C**) (see ProteomeXchange under the project accession PXD046029 for the detail), indicating cytoplasmic protein contaminations. This may because the cell walls of gram-negative bacteria were more prone to lysis during trypsinization (37), which reflected a limitation of the bacterial surface shaving technique. Therefore, we used membrane-impermeable biotin technique to specifically label bacterial surface proteins, which were then enriched with streptavidin-agarose and analyzed via LC-MS/MS (**Figures S2D, S2E**) (15). 967 potential surface-exposed proteins were identified (**Figure S2F**) (see ProteomeXchange under the project accession PXD046029 for the detail), which accounted for 44.6% HBS1 proteins and far exceed the reasonable proportion of membrane proteins (38). This may because small amounts of biotin were able to enter the cell and label the cytoplasmic proteins, which were then enriched along with surface proteins (15). To overcome these challenges, we developed a pipeline to identify surface-associated proteins by a combination of *in situ* biotinylation, TPCK-trypsin digestion, streptavidin-agarose pulldown, and LC-MS/MS analysis (**Figure 1C**). The HBS1 cells were first *in situ* biotinylated. After rounds of washes to remove excessive biotin and cell lysate, biotin-labelled surface-exposed proteins were released from intact bacterial cells by partial digested with TPCK-treated trypsin and identified by mass spectrometry analysis after streptavidin-agarose pulldown. A total of 333 unique proteins (**Table S7**) were identified, accounting for 15% of the HBS1 genome. Most important, only 31 proteins were identified in the control group with 18 proteins overlapping the treat group, indicating the less contamination of cytoplasmic proteins compared to surface-shaving and membrane-impermeable biotin techniques (**Figures 1C**, **S2**). These results demonstrated an optimal pipeline to identify bacterial surface-exposed proteins by combing use of *in situ* biotinylation, TPCK-trypsin digestion, streptavidin-agarose pulldown, and mass spectrometry analysis.

To construct a T7 phage library for BacScan, genes encoding surface-exposed proteins, predicted secreted proteins, and reported immunogenic antigens (38–46) were individually split into 600-bp equal-length DNA fragments with a 300-bp overlap between adjacent fragments (**Figure 1D**). After removing the overlap proteins between secreted proteins, surface-associated proteins, and reported immunogenic antigens (**Figure 1D**, Venn diagram), a total of 445 genes (1,359 DNA fragments) were cloned into T7 phage genome to generated a T7 display library. The quality of the BacScan library was assessed via Illumina sequencing to a depth of 50-fold coverage, and 80.8% of the inserts were present in the library (**Figure 1E**) (see the Sequence Read Archive (SRA) under BioProject accession number PRJNA1026755 for the detail).

### Identification of highly immunogenic proteins of *A. pleuropneumoniae* using BacScan

**Figure 2:**
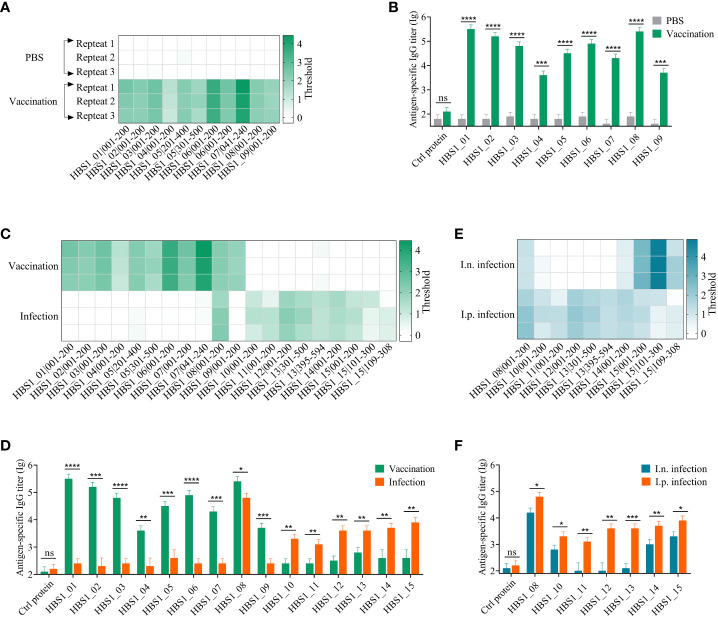
Identification of HIPs of *A*. *pleuropneumoniae* using BacScan. **(A)** Identification of HIPs using sera prepared by immunizing mice with inactivated *A*. *pleuropneumoniae* HBS1. Three parallel experiments were carried out for each serum sample, and each row represents one repetition. The enriched gene fragments were shown at the bottom. The color intensity of each cell indicates the threshold for enrichment. **(B)** ELISA results show the binding of identified HIPs to *A*. *pleuropneumoniae*-specific sera. Influenza viral 3M2e protein was used as a negative control. **(C)** Comparison of HIPs identified using vaccine sera and infection sera. Serum samples were prepared as described in the Materials and Methods. HIPs were identified using BacScan. **(D)** The binding of 15 HIPs to vaccine sera and infection sera. ELISA plates were coated with HIPs individually, and influenza viral 3M2e protein was used as a negative control. **(E)** Comparison of HIPs identified using intranasal (i.n.) and intraperitoneal (i.p.) infection sera. **(F)** The reactivity of 7 HIPs to i.n. and i.p. infection sera. Influenza viral 3M2e protein was used as a negative control. Data are shown as means ± S.D. *, **, *** and **** indicate p < 0.05, p < 0.01, p < 0.001 and p < 0.0001, respectively (Student’s t-test).

To avoid cross-immune reaction with other bacteria, we first prepared *A. pleuropneumoniae*-specific sera using specific-pathogen free (SPF) mice, which were immunized intraperitoneally with inactivated *A. pleuropneumoniae* HBS1 on days 2, 16, and 23 (**Figure S3A**). Control mice were given the same volume of PBS. The titers of *A. pleuropneumoniae*-specific antibodies were significant increased after each boost (**Figure S3B**). Seven days after the last immunization, sera were collected and incubated with T7 library for BacScan analysis. The phage-antibody complexes were precipitated using protein A/G magnetic beads, and the enriched phages were amplified by PCR and sequenced to determine the inserts (17). 11 unique fragments belong to 9 proteins (**Table S5**) were enriched in all three replicates (**Figure 2A**), indicating that the method was highly reproducible.

To assess the specificity and sensitivity of BacScan, nine full-length proteins were expressed and purified from *Escherichia coli* cells (**Figure S4A**). Influenza viral protein 3M2e, which was expressed and purified from *E. coli* in our previous study (47), was used as a negative control. Indirect ELISA results showed that the sera from immunized mice exhibited specific reactivity towards nine HIPs but not towards the 3M2e control protein (**Figure 2B**). Furthermore, the preimmunized sera did not show any reactivity towards these proteins. These results indicated that BacScan is a powerful tool for identifying HIPs.

To determine whether the antigenic targets of humoral immune responses in mice differ between inactive bacterial vaccination and bacterial infection, each mouse was infected intraperitoneally with 2.5×10^6^ CFU *A. pleuropneumoniae* HBS1 to prepare infection sera. The ELISA results showed that the titer of *A. pleuropneumoniae*-specific IgG is low 7 days after infection, which was significantly boosted after two more rounds of infections (**Figure S3C**). Therefore, the sera after third infection were used for BacScan analysis. Interestingly, among 10 fragments belonging to 7 unique proteins (**Table S5**) enriched in the infection group, only one fragment (HBS1_08|001-200) was enriched in the vaccination group (**Figure 2C**). To further confirmed the difference, we expressed and purified 6 differentially enriched proteins from *E. coli* cells (**Figure S4B**) to determine their reactivities against sera from the vaccination group and the infection group. ELISA data showed that HBS1_08 protein can be recognized by sera from both groups, whereas all the 6 differentially enriched proteins showed high reactivities only against sera from the infection group but not from the vaccination group and vice versa (**Figure 2D**).

Although peritoneal inoculation is the primary route of infection used in mouse models (48), the natural route of infection for *A. pleuropneumoniae* is the respiratory tract. Therefore, we compared the antigenic differences of *A. pleuropneumoniae* between intranasal (i.n.) and intraperitoneal (i.p.) infections. After the third-round i.n. infection of mice (**Figure S3D**), sera were collected for BacScan analysis. Interestingly, all the 5 fragments belonging to 3 unique proteins enriched in the i.n. infection group were enriched in the i.p. infection group. The fragments HBS1_10|001-200 and HBS1_14|001-200 showed weak binding in the i.n. infection group (**Figure 2E**). ELISA data also showed these 4 HIPs can be recognized by sera from both groups, while the other three proteins (HBS1_11, HBS1_12, and HBS1_13) enriched only in the i.p. infection group showed background binding activities against sera from the i.n. infection group (**Figure 2F**). These results indicated that immunization and infection, even different infection routes, might induce the production of an antibody repertoire different from each other, which can be detected by BacScan technology.

### Identification of target antigens for the development of serological diagnostics for *A. pleuropneumoniae*

**Figure 3:**
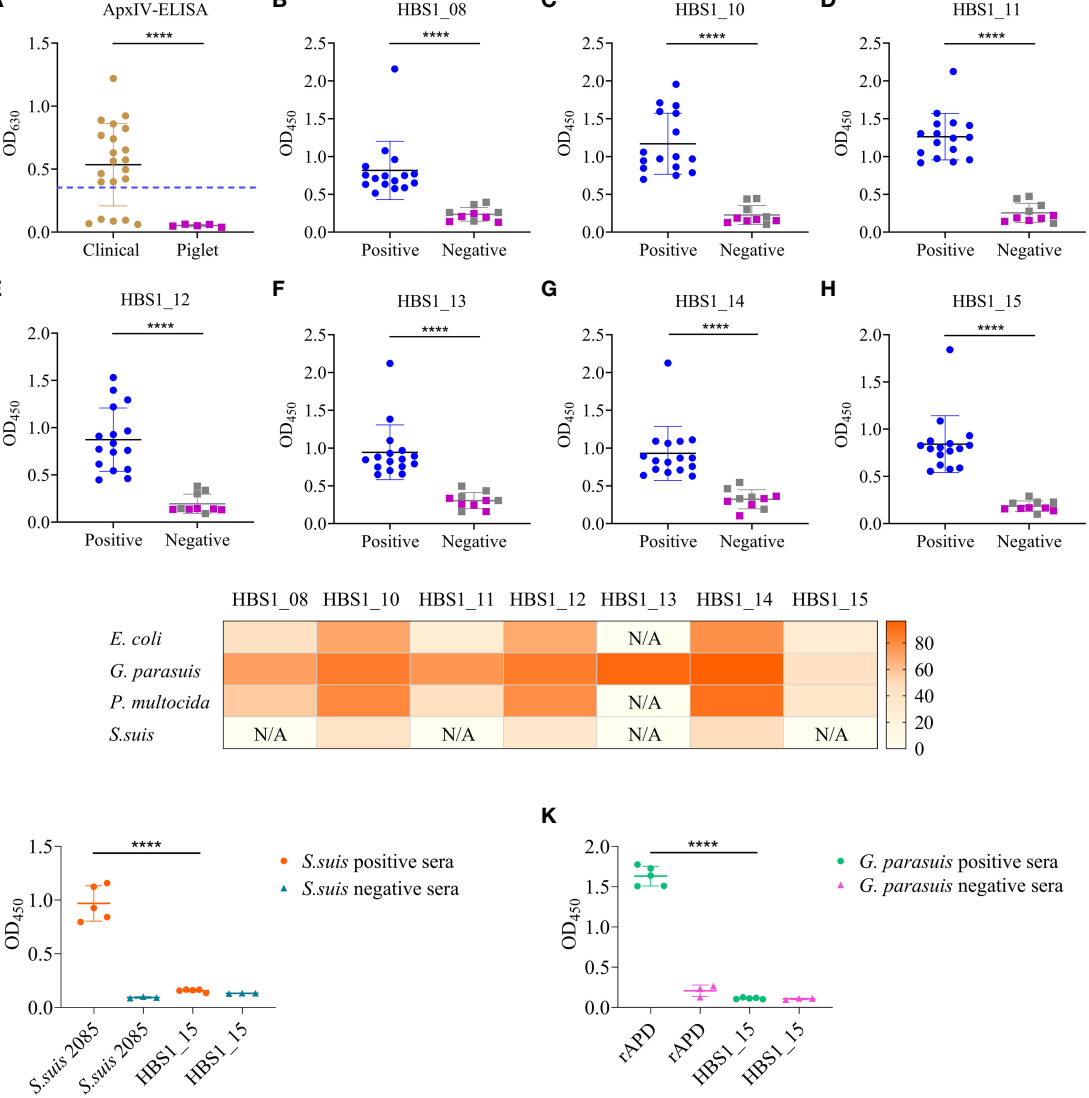
Identification of target antigens for serological diagnostics of *A*. *pleuropneumoniae.* **(A)** Detection *A*. *pleuropneumoniae* positive sera using the ApxIV ELISA kit. Dash line represents the threshold of the kit. **(B–H)** ELISA results showing the binding activities of the 7 HIPs to swine sera. Gray squares represent *A*. *pleuropneumoniae* negative clinical sera and purple squares represent piglet sera. **(I)** Homology analysis of 7 A*. pleuropneumoniae* HIPs in four common bacterial pathogens of pigs. The color intensity of each cell indicates the homology (%) of 7 A*. pleuropneumoniae* HIPs of in other bacterial species. “N/A” indicates not detected. **(J, K)** Cross-reactivities of *A*. *pleuropneumoniae* HBS1_15 to *S. suis* positive sera **(J)** and *G. parasuis* positive sera **(K)**. The recombinant *S. suis* 2085 protein was used as a control to confirm the *S. suis* positive and negative sera. The *G. parasuis* rAPD protein was used as a control to confirm the *G. parasuis* positive and negative sera. Data are shown as means ± S.D. **** indicates p < 0.0001 (Student’s t-test).

The HIPs identified from the infection groups but not the immunization group could be targets for the development of differential diagnostics that can distinguish between vaccine immunization and natural infection. To determine any of the 7 proteins identified from the i.p. infection could be targets for the development of serological detection methods, 21 pig sera were randomly collected from different farms. Five piglet sera negative for common swine pathogens prepared in our previous study were used as controls. Among these, 16 sera are *A. pleuropneumoniae* positive determined using a commercial *A. pleuropneumoniae* ApxIV ELISA kit (**Figure 3A**). The sera were then individually tested for their binding activities to the 7 HIPs. The ELISA results showed that all 7 proteins had strong binding activities to the 16 positive pig sera but not to the 5 piglet negative sera, which was consistent with the results of the commercial kits (**Figures 3B–H**). However, some of the 5 A*. pleuropneumoniae-*negative clinical sera showed weak binding activities to the HIPs compared to the 5 piglet sera, which may be due to the cross-reaction of these HIPs with other pathogen-specific sera (see below).

To determine the specificity of these proteins as targets for detecting *A. pleuropneumoniae*, seven proteins were used individually to Blast the homologous proteins from common bacterial pathogens of pigs, *E. coli*, *Glaesserella parasuis*, *Pasteurella multocida*, and *S. suis*. Overall, all these proteins showed higher sequence similarity to homologous proteins from *G. parasuis* but lower similarity to homologous proteins from *S. suis* (**Figure 3I**). Significantly, we found that HBS1_15 had very low sequence similarity (28-45%) to the homologous proteins from four common bacterial pathogens, indicating its potential as a specific target for *A. pleuropneumoniae* detection. To further verify its specificity, HBS1_15 proteins were coated onto ELISA plates to evaluate its reactivity with *S. suis* or *G. parasuis* positive swine sera. The results demonstrated that HBS1_15 did not show cross-reactivity (**Figures 3J, K**). Taken together, these results suggest that the HBS1_15 protein is a promising target for the development of serological diagnostic methods for *A. pleuropneumoniae*.

### Identification of protective antigens of *A. pleuropneumoniae*

To avoid omissions, all the 15 proteins (**Table S5**) identified using different sera were included in our analysis to identify protective of *A. pleuropneumoniae*. We first performed sequence analysis to determine which of the 15 candidate antigens are conserved among different serotypes. Sequence BLAST revealed that 14 of the 15 proteins are highly conserved among different serotypes, while the HBS1_13 protein is only conserved among serotypes 1, 6, 12, 14, and 17, and not with other serotypes (**Figure S5A**). HBS1_01, and HBS1_07 have more than 30% homology with porcine proteins (**Figure S5B**) and were therefore excluded from the animal experiment to avoid the potential autoimmunity. Two of the 15 proteins, namely HBS1_11 (49) and HBS1_13 (40), were previously identified as protective antigens and were therefore used as positive controls in our animal experiment. In addition, HBS1_03 protein, which was found to be immunogenic but not protective, was excluded from our animal experiments (42).

**Figure 4:**
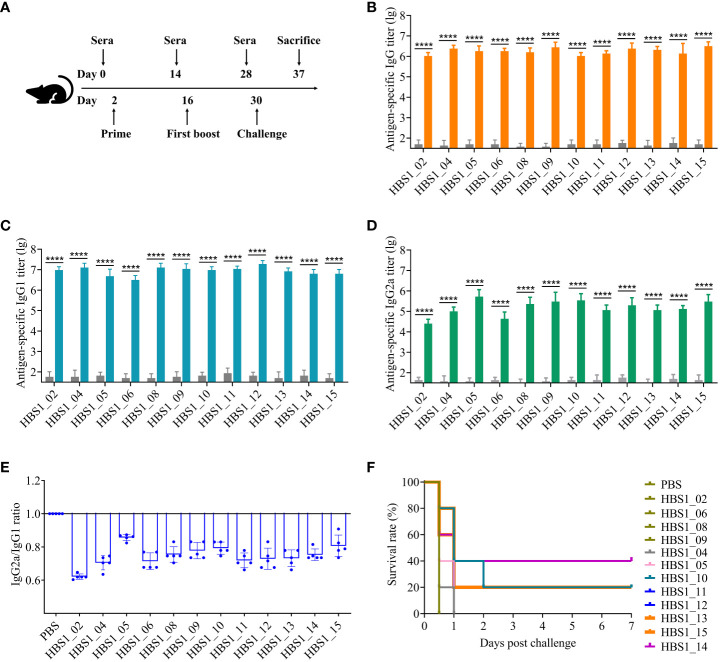
Identification of protective antigens of *A*. *pleuropneumoniae*. **(A)** Experimental scheme for immunization and challenge of mice. Sera were collected two weeks after each immunization. HIP-specific IgG **(B)**, IgG1 **(C)**, and IgG2a **(D)** titers were determined using ELISA. **(E)** The ratio of the IgG2a to IgG. Data are shown as means ± S.D. **** indicates p < 0.0001 (Student’s t-test). **(F)** Survival rate of mice. Mice were challenged with 2.5 × 10^7^ CFU of *A*. *pleuropneumoniae* HBS1 two weeks after the last immunization and monitored for 7 days. The survival curves were analyzed by log-rank test. Significant differences were observed between HBS1_10/13/14/15 and PBS in survival rate (log-rank test, P < 0.05).

To investigate the immunoprotective effects of the 12 candidate antigens, the recombinant proteins were emulsified with adjuvant ISA 201 and injected intramuscularly into mice, individually, on day 2 and day 16 (**Figure 4A**). Sera were collected for ELISA analysis of the humoral immune responses, and the results showed that all 12 proteins induced higher levels of IgG antibodies compared to the PBS control group (**Figure 4B**). All antigens were able to induce both IgG1 (Th2-biased) and IgG2a (Th1-biased) antibodies. However, IgG1 titers are higher than IgG2a titers for each of 12 antigens (**Figures 4C–E**), suggesting that these recombinant proteins in combination with adjuvant ISA 201 mainly induce Th2-biased immune responses.

The protective efficacy of each antigen was determined by challenge of vaccinated mice with 2.5 × 10^7^ CFU of *A. pleuropneumoniae* HBS1 two weeks after boost. Interestingly, of the two previously identified protective antigens (HBS1_11, and HBS1_13) (40, 49), HBS1_13 can induce 20% protection against challenge (**Figure 4F**; p = 0.0143; log-rank test). This might be because of the high virulence of *A. pleuropneumoniae* HBS1. Nevertheless, we found that three new antigens, namely HBS1_14, HBS1_10, and HBS1_15 could still provide 40% (p = 0.0495; log-rank test), 20% (p = 0.0143; log-rank test), and 20% (p = 0.0495; log-rank test) protection individually. As shown in **Figure 4F**, all mice in the groups of PBS control, HBS1_02, HBS1_04, HBS1_05, HBS1_06, HBS1_08, HBS1_09, HBS1_11, and HBS1_12 died 12 hours after challenge. These results suggest that HBS1_14, HBS1_10, and HBS1_15 are potential targets for the development of *A. pleuropneumoniae* subunit vaccines.

### A multivalent subunit vaccine containing HBS1_14 and toxin proteins enhances the immune protection against *A. pleuropneumoniae*

**Figure 5:**
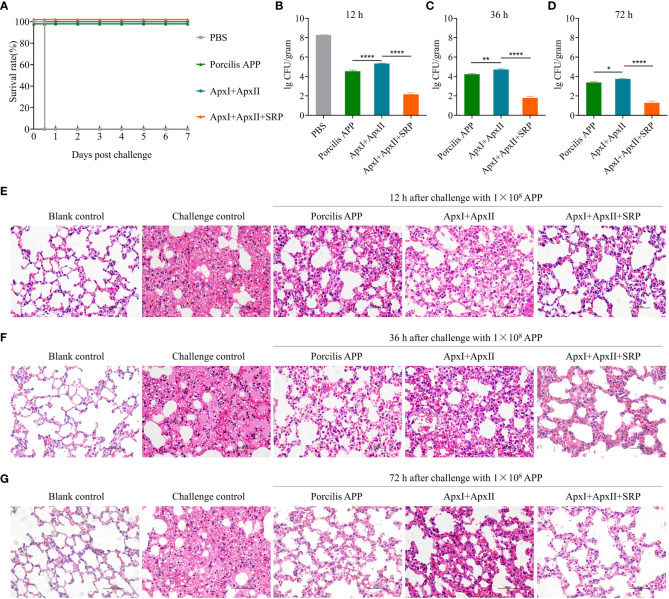
A multivalent subunit vaccine containing HBS1_14 (SRP) and toxin proteins enhances the immune protection against *A*. *pleuropneumoniae*. **(A)** Survival rate of mice after challenge with 1.0 × 10^8^ CFU of *A*. *pleuropneumoniae* HBS1. The bacterial loads in lung tissues were determined at 12 **(B)**, 36 **(C)**, and 72 **(D)** hours after challenge. Data are presented as means ± S.D. *, **, and **** represent p < 0.05, p < 0.01, and p < 0.0001 (ANOVA). Pathological changes of mouse lung tissues were determined at 12 **(E)**, 36 **(F)**, and 72 **(G)** hours after challenge (scale bar, 50 μm). The naïve mice challenged with the same dose of *A*. *pleuropneumoniae* HBS1 were used as challenge controls.

The highly pathogenic serotype 1 HBS1 strain secretes both ApxI and ApxII toxins, which belong to pore-forming repeat-in-toxins (RTXs) and are the critical virulent factors of *A. pleuropneumoniae* (50). We hypothesize that toxin-specific antibodies will neutralize the toxin and alleviate the disease caused by the toxin, while HBS1_14-specific antibodies will help eliminate the bacteria. *A. pleuropneumoniae* subunit vaccines that can induce both toxin-specific and 14-specific antibodies should be more effective than either alone. Therefore, we want to investigate whether a multivalent subunit vaccine containing HBS1_14 (Signal recognition particle, SRP), ApxI, and ApxII (**Figure S4C**) could induce enhanced immune protection. Mice were immunized intramuscularly twice, two weeks apart, with ApxI+ApxII+SRP (**Figure S6A**). Mice immunized with ApxI+ApxII, commercial vaccine (Porcilis APP, containing ApxI, II, III, and one membrane protein), or PBS were used as controls. ELISA results showed that all mice immunized with antigens generated robust ApxI-specific and ApxII-specific IgG in sera two weeks after last vaccination (**Figure S6B, C**). SRP-specific IgG was observed only in ApxI+ApxII+SRP (**Figure S6D**). As expected, mice in the PBS group generated only background levels of antigen-specific IgG. Protective efficacy was determined by challenging the vaccinated mice with a high dose (1.0 × 10^8^ CFU) of *A. pleuropneumoniae* HBS1 strain two weeks after boost. All mice in the PBS group died 12 hours after challenge, whereas all mice in the Porcilis APP, ApxI+ApxII, and ApxI+ApxII+SRP groups survived till sacrifice 7 days after challenge (**Figure 5A**), indicating that ApxI and ApxII together were able to provide complete protection.

To further determine whether the inclusion of SRP enhances the protective efficacy, 9 mice were immunized with ApxI+ApxII+SRP and challenged as shown in **Figure S6A**. Three mice were sacrificed at 12-, 36-, and 72-hours post challenge, respectively. Lungs were harvested for bacterial load determination and pathological analysis. Mice immunized with Porcilis APP or ApxI+ApxII were used as controls. The results showed that the lung bacterial load in the ApxI+ApxII+SRP group was approximately 1×10^1~2^ CFU/gram lung tissue, which is significantly lower than ApxI+ApxII group. Similarly, the lung bacterial load in the Porcilis APP group was significantly lower than that in the ApxI+ApxII group (**Figures 5B–D**). In addition, pathological analysis showed that mice in the ApxI+ApxII group exhibited thickening of the alveolar walls and widening of the pulmonary interstitium, whereas the ApxI+ApxII+SRP group showed only mild pathological changes (**Figures 5E–G**). As expected, blank control mice showed no obvious pathological changes, whereas naive mice challenged with *A. pleuropneumoniae* HBS1 exhibited severe pathological changes. Mice in the commercial vaccine (Porcilis APP) group also showed fewer pathological changes compared to the ApxI+ApxII group, but similar to the ApxI+ApxII+SRP group (**Figures 5E–G**). These results suggest that the inclusion of SRP (HBS1_14) may enhance the protective efficacy of ApxI+ApxII based subunit vaccines.

## Discussion

Emerging viral and bacterial epidemics, as exemplified by SARS-CoV-2 and methicillin-resistant *Staphylococcus aureus* (MRSA), pose a significant threat to both human and animal populations (51, 52). Due to the diversity of bacterial serotypes and the limited cross-protection offered by current bacterial vaccines (53), there is an urgent need for standardized methods to expedite the development of antigen-matched vaccines to prevent the spread of emerging novel bacteria or serotypes. Rapid *de novo* identification of protective antigens from a clinical isolate represents the first and critical step in the development of effective subunit vaccines against circulating bacterial strains. In this study, we have developed a universal pipeline that combines proteosurfaceomics, secretomics, and BacScan technology to rapidly identify bacterial protective proteins *de novo* from a clinical isolate without prior knowledge of the pathogenic bacterium. Our strategy is particularly suitable for emerging novel pathogenic bacteria or serotypes.

While numerous high-throughput methods are capable of identifying immunogenic proteins, their application to the *de novo* identification of high immunogenic proteins (HIPs) from uncharacterized bacteria is limited. For example, reverse vaccinology is a powerful method that requires numerous complete genome sequences, which may not be available for uncharacterized bacteria, to identify candidate antigenic proteins (11). Protein array does not require numerous genome sequences, but the cost of expressing all bacterial proteins is considerable (54). Traditional phage display involves multiple rounds of selection, which may introduce bias toward phages with higher propagation rates (55). The approach of combining two-dimensional SDS-PAGE of bacteria with western blot using bacterial-specific sera disrupts the structure of native proteins, thereby affecting the screening results (56). Our method, which takes advantage of proteosurfaceomics, secretomics and BacScan technologies, allows rapid *de novo* identification of protective antigens from an uncharacterized novel bacterium. Once the pathogenic strain was identified, the genome was sequenced to subsequently identify secreted or surface-associated proteins, whose genes were amplified by PCR to rapidly construct a T7 phage display library using a commercial kit. The T7 phages bound to the bacterial-specific sera are enriched by a single round of selection, allowing rapid identification of all HIPs by next-generation sequencing.

As a proof of concept, we isolated a highly virulent *A. pleuropneumoniae* strain from a clinical sample and identified 15 HIPs using this technology. All 15 HIPs showed high immunoreactive activity with *A. pleuropneumoniae*-specific sera (**Figures 2B, D, F**), and three of them have been reported in previous studies (40, 42, 49). These results demonstrate the specificity and reliability of our method. In particular, we found that HBS1_15, which is highly conserved among different *A. pleuropneumoniae* serotypes but has low homology with proteins from common bacterial pathogens of pigs (**Figures 3I**, **S5A**), has great potential as a diagnostic target. Significantly, we identified three novel protective antigens, HBS1_10, HBS1_14, and HBS1_15, which individually can provide partial protection against challenge in mice (**Figure 4F**). Importantly, we showed that a multivalent subunit vaccine containing HBS1_14 (SRP) and toxin proteins provided complete protection against lethal dose of *A. pleuropneumoniae* challenge in mice, with enhanced immune protection compared to toxin proteins alone (**Figure 5**).

In addition, we have developed a highly specific method for identifying surface-associated proteins by combining *in situ* biotinylation, TPCK-trypsin shaving, streptavidin-agarose pulldown, and mass spectrometry (**Figure 1C**). Bacterial surface shaving is a recently developed technique to release surface-exposed proteins from bacterial cells by partial cleavage with proteolytic enzymes such as trypsin, which are then detected by mass spectrometry (14, 27). However, we observed that this method resulted in a significant background signal (**Figures S2A–C**). This may be due to the susceptibility of Gram-negative bacterial cell walls to lysis during trypsinization, as previously reported (14, 16). The membrane-impermeable biotin technique selectively labels bacterial surface proteins with biotin, which are then enriched with streptavidin-agarose for subsequent mass spectrometry after bacterial cell lysis (15). However, we have observed that the cytoplasmic proteins can still be labeled either by residual biotin or by small amounts of biotin entering the cells, resulting in a high background signal (**Figures S2D–F**). Our combined strategy overcomes the limitations of both methods. After biotinylation, the biotin-labeled surface proteins were released from intact bacterial cells by TPCK-trypsin shaving instead of lysing the cells, thus significantly reducing cytoplasmic protein contamination. Our strategy not only increases the screening efficiency of BacScan, but can also be used to identify bacterial surface proteins for other purposes.

Although our strategy can rapidly identify bacterial protective antigens *de novo*, the results of the screen are highly dependent on the quality of the sera. On the one hand, the chance of identifying protective antigens is higher if the antibodies in the sera have high antimicrobial activity. Indeed, we found that different HIPs were identified between vaccinated and bacterium infected sera (**Figure 2C**). Even the route of infection affects the results of the screen (**Figure 2E**), suggesting the importance of sera quality. On the other hand, cross-reactivity of antibodies between different bacteria may interfere with the results of the screen. Therefore, sera must be prepared using specific pathogen-free (SPF) animals, and the bacterial strain used for infection must express all proteins critical for its infection. This is also the reason why we used SPF mice to prepare *A. pleuropneumoniae*-specific sera rather than using clinically positive swine sera. However, it is difficult to know whether all the proteins critical for its infection are expressed when cultured *in vitro*. This can be overcome by using sera prepared with bacterial cells cultured under different conditions. Nevertheless, our strategy may not identify all the protective antigens, but the most critical ones.

In conclusion, we have developed a universal pipeline integrating proteosurfaceomics, secretomics, and BacScan technologies for the rapid *de novo* identification of bacterial protective proteins from clinical isolates. Using this technology, we identified 12 novel antigenic proteins of *A. pleuropneumoniae*. Among these proteins, HBS1_15 shows great potential as a serologic diagnostic target, whereas HBS1_14 is a promising vaccine target. Using HBS1_14 and toxin proteins, we have developed a promising multivalent subunit vaccine against *A. pleuropneumoniae*. We believe that our strategy can be applied to any bacterial pathogen and has the potential to significantly accelerate the development of antigen-matched vaccines, thereby preventing the spread of emerging novel bacteria or serotypes.

## Data availability statement

The original contributions presented in the study are included in the article/**Supplementary Material**. Further inquiries can be directed to the corresponding author.

## Ethics statement

The animal study was approved by Research Ethics Committee of Huazhong Agricultural University (Approval number: HZAUMO-2021-0130). The study was conducted in accordance with the local legislation and institutional requirements.

## Author contributions

JY: Data curation, Methodology, Project administration, Validation, Writing – original draft, Writing – review & editing. XZ: Validation, Writing – original draft, Writing – review & editing. JD: Validation, Writing – original draft, Writing – review & editing. QZ: Methodology, Writing – original draft, Writing – review & editing. ES: Methodology, Writing – original draft, Writing – review & editing. CC: Validation, Writing – original draft, Writing – review & editing. ZM: Validation, Writing – original draft, Writing – review & editing. YZ: Validation, Writing – original draft, Writing – review & editing. NZ: Validation, Writing – original draft, Writing – review & editing. PT: Funding acquisition, Writing – original draft, Writing – review & editing.
