# Supplementary material for: De novo identification of bacterial antigens of a clinical isolate by combining use of proteosurfaceomics, secretomics, and BacScan technologies
Source: Front Immunol. 2023 Nov 30;14:1274027. doi: 10.3389/fimmu.2023.1274027 (PMC10720918; doi:10.3389/fimmu.2023.1274027)
Supplement: Supplementary file 1 [file DataSheet_1.docx]

**Supporting information**

**De novo identification of bacterial antigens of a clinical isolate by combining use of proteosurfaceomics, secretomics, and BacScan technologies**

Jinyue Yang ^1,2,3^, Xueting Zhang ^1,2,3^, Junhua Dong ^1,2,3^, Qian Zhang ^1,2,3^, Erchao Sun ^1,2,3^, Cen Chen ^1,2,3^, Zhuangxia Miao ^1,2,3^, Yifei Zheng ^4^, Nan Zhang ^5^, and Pan Tao ^1,2,3*^.

^1^ State Key Laboratory of Agricultural Microbiology, College of Veterinary Medicine, Huazhong Agricultural University, Wuhan, Hubei 430070, China.

^2^ Key laboratory of Prevention & Control for African Swine Fever and Other Major Pig Diseases, Ministry of Agriculture and Rural Affairs. Cooperative Innovation Center for Sustainable Pig Production, Huazhong Agricultural University, Wuhan, Hubei 430070, China.

^3^ Hubei Hongshan Lab, Wuhan, Hubei 430070, China.

^4^ Veterinary diagnostic laboratory, Neixiang center for animal disease control and prevention, Nanyang, Henan 473000, China.

^5^ Neixiang animal health supervision, Neixiang animal husbandry bureau, Nanyang, Henan 473000, China.

*To whom correspondence should be addressed. E-mail: taopan@mail.hzau.edu.cn

**Supplementary Figure 1**

**
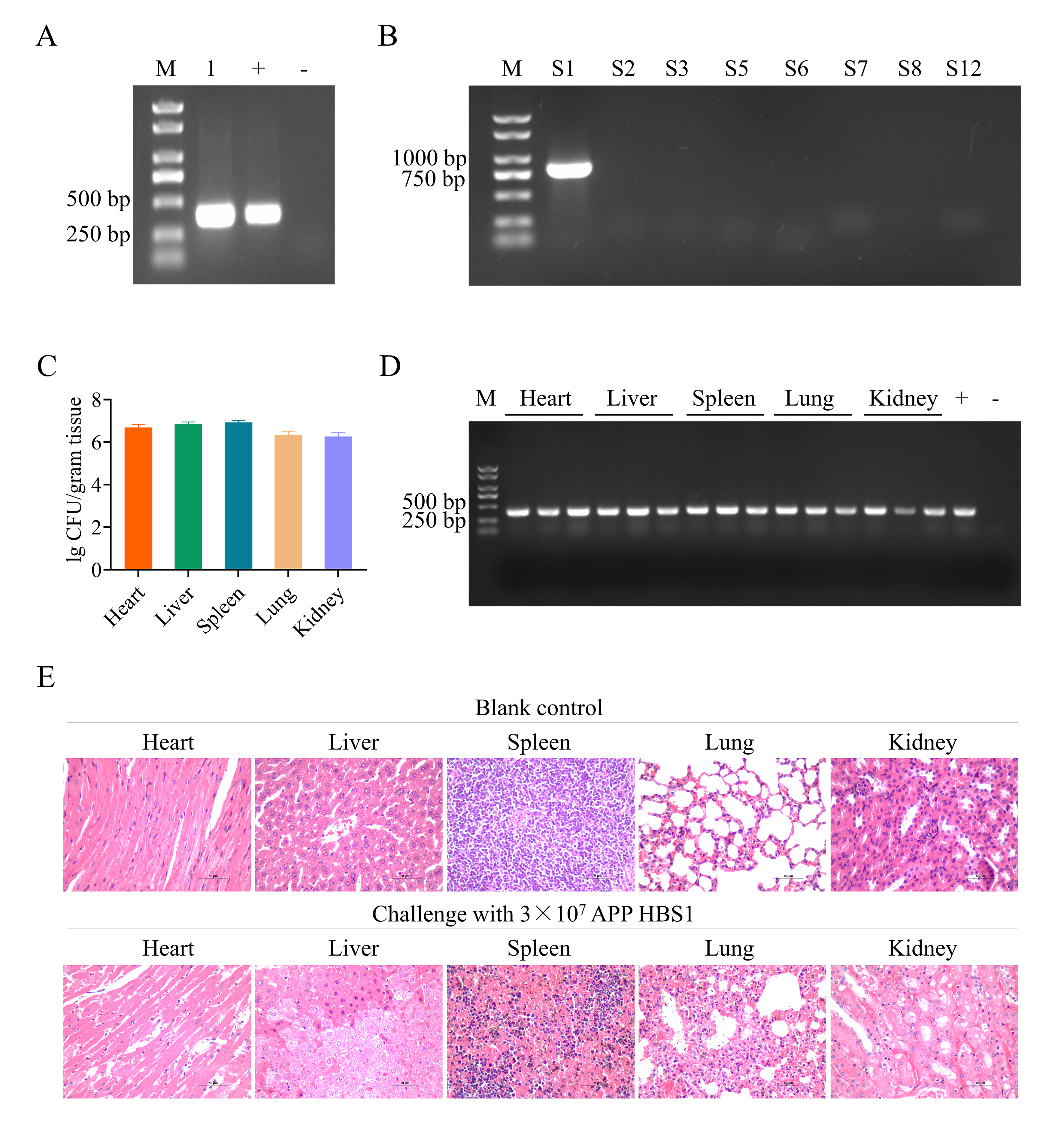
**

**Figure S1 Identification and pathogenicity of APP HBS1 strain.** PCR (A) and serotype (B) identification of the isolate. M, DL 2000 DNA marker. "1" represents the isolated strain, "+" represents positive control for *A. pleuropneumoniae*, and "-" represents the negative control. S1-S12 correspond to specific primers for identifying different serotypes of *A. pleuropneumoniae*. (C) Bacterial load assay in the heart, liver, spleen, lung and kidney of mice (n=3). After 12 hours post-infection, a portion of tissues was weighed, ground, filtered, and subjected to bacterial load analysis. The results showed that the bacterial loads in these tissues of mice reached 10^6^ CFU/g. (D) PCR identification results of bacteria isolated from each tissue. Three single bacterial colonies were randomly selected from each tissue and identified by PCR, all of which were confirmed to be *A. pleuropneumoniae*. (E) Histopathological analysis of different tissues in both blank control and infected (challenge with 3×10^7^ CFU APP HBS1) mice using HE staining (scale bar, 50 μm). No significant pathological changes were observed in the hearts of mice compared to the blank control, while the other tissues showed varying degrees of pathological changes.

**Supplementary Figure 2**





**Figure S2 Identification of APP surface proteins.** (A) Flowchart illustrating the identification of APP surface proteins through TPCK-Trypsin digestion and mass spectrometry analysis. (B) Efficiency of TPCK-Trypsin in removing surface-exposed proteins. SDS-PAGE analysis of "surfome" fractions stained with Coomassie Brilliant Blue at different digestion times, expressed in minutes ("-" denotes no TPCK-Trypsin treatment, while "+" denotes TPCK-Trypsin treatment). (C) Venn diagram illustrating the overlap of surface proteins enriched in the TPCK-Trypsin digestion compared to the control preparation. Ctrl refers to the control preparation without TPCK-Trypsin treatment. (D) Flowchart depicting the procedure for enriching APP surface proteins using biotin-streptavidin and mass spectrometry analysis. (E) Efficiency of biotin labeling for surface-exposed proteins. Western blot analysis of surface proteins labeled with biotin at various time points, measured in minutes. (F) Venn diagram showing the overlap of surface proteins enriched through biotin-streptavidin compared to the control preparation. Ctrl indicates the control preparation without biotin labeling.

**Supplementary Figure 3**


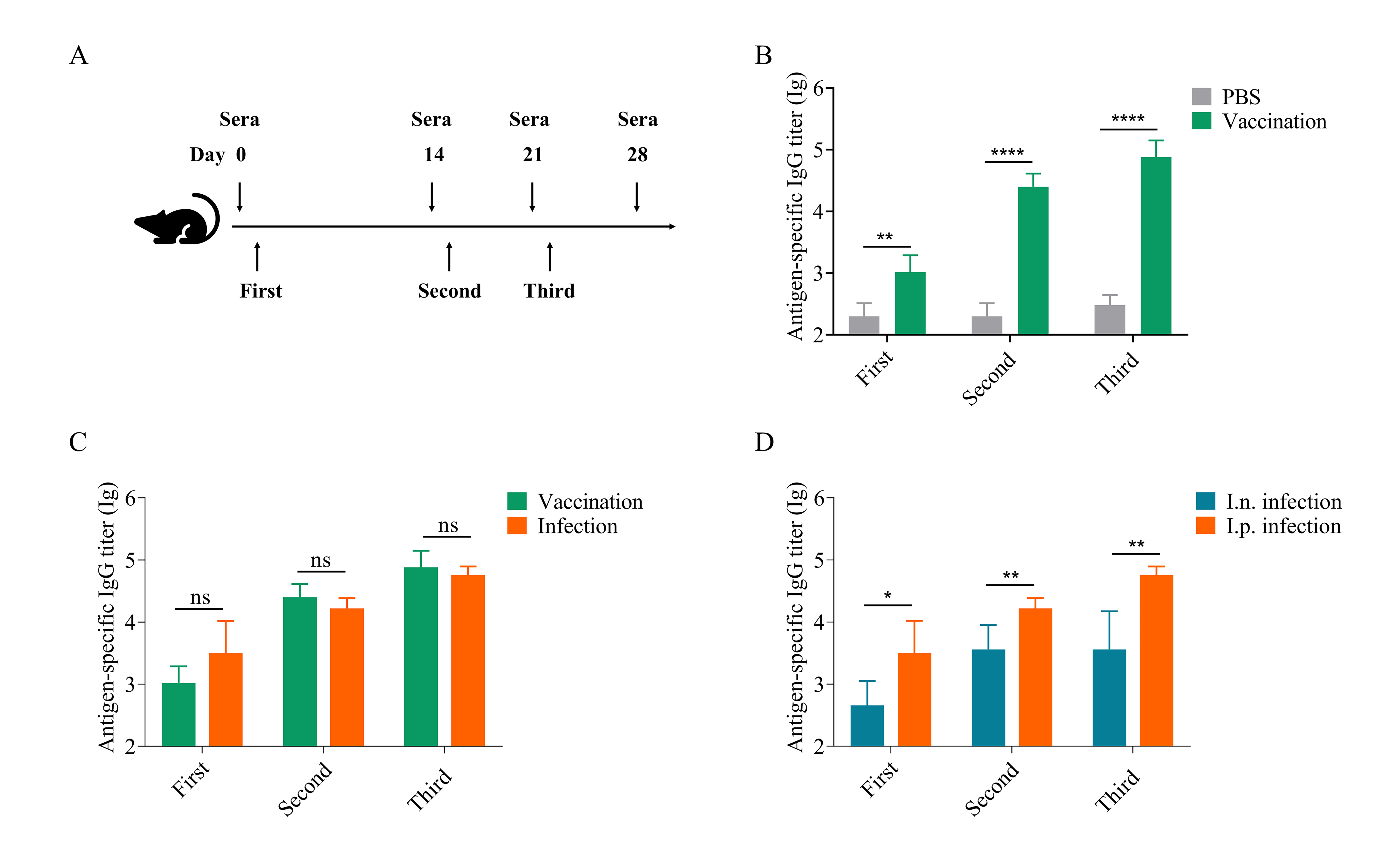


**Figure S3 Titration ELISA to determine mice sera following immunization or infection.** (A) Schematic diagram of the immunization or infection procedure. (B) Determination of specific anti-APP antibody titers in the sera of mice immunized with the inactivated vaccine at 14, 21, and 28 days. (C) Comparative analysis of antibody titers in mice sera between vaccination and infection. (D) Comparative analysis of antibody titers in mice sera between intranasal (i.n.) and intraperitoneal (i.p.) infections. Data are shown as means ± S.D. * represents p < 0.05, ** represents p < 0.01, **** represents p < 0.0001, and ns represents no significant difference (p > 0.05) (Student's t-test).

**Supplementary Figure 4**


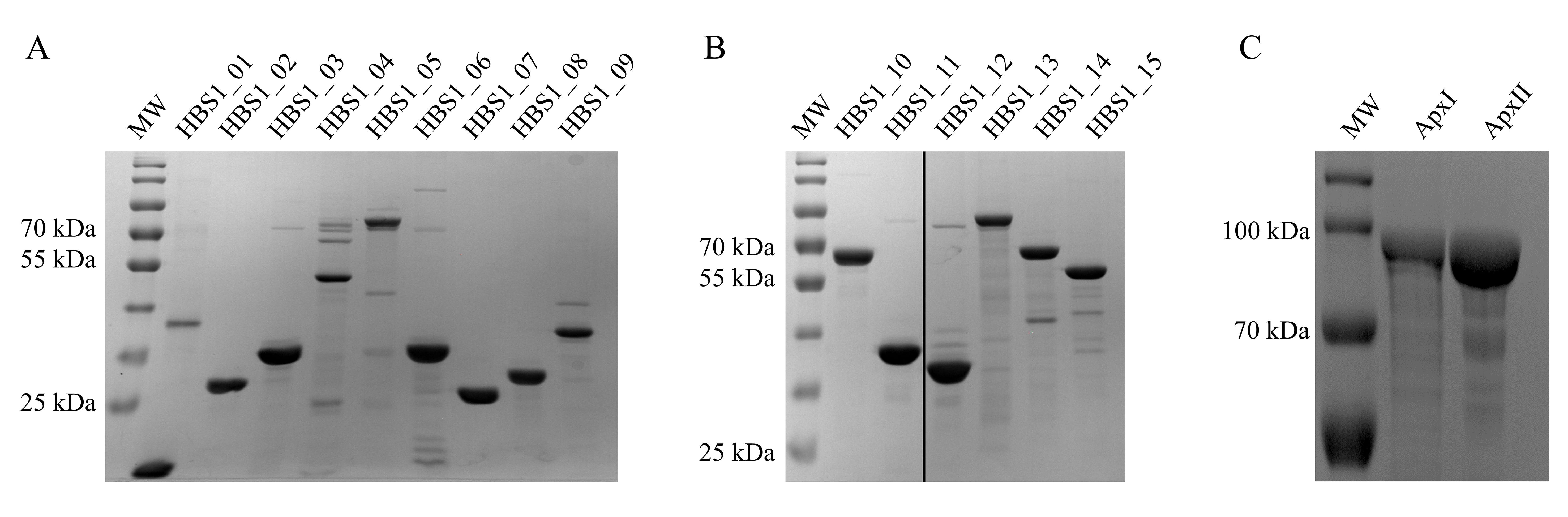


**Figure S4 Expression and affinity purification of recombinant proteins.** (A) SDS-PAGE analysis of nine purified recombinant proteins. (B) SDS-PAGE analysis of six purified recombinant proteins. (C) SDS-PAGE analysis of purified toxin proteins. MW, standard protein marker.

**Supplementary Figure 5**


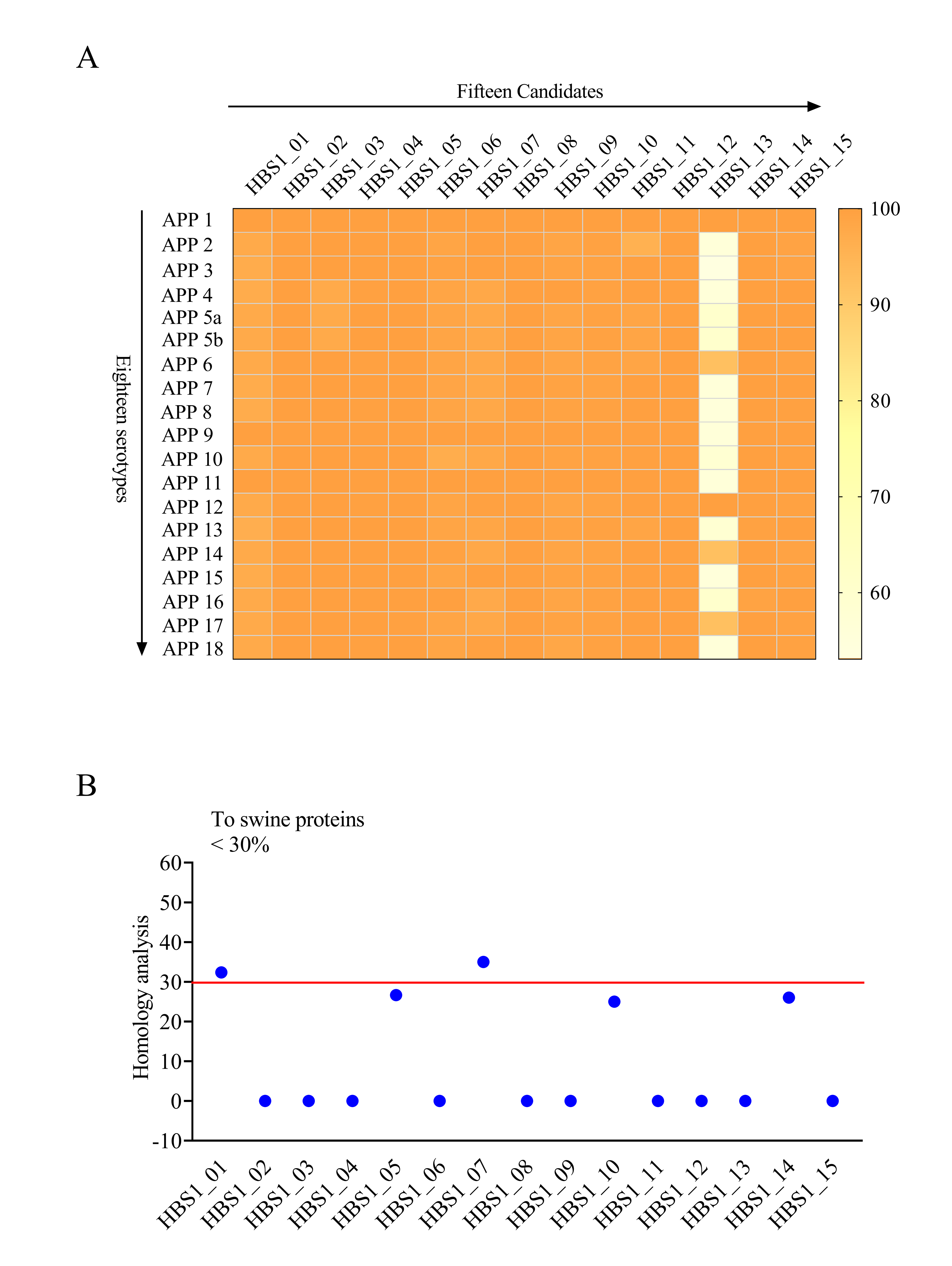


**Figure S5 Homology analysis of 15 proteins using BLAST.** (A) Homology analysis of 15 proteins across 18 serotypes of *A. pleuropneumoniae*. Each row represents a serotype of *A. pleuropneumoniae*, and each column represents a candidate protein. The color intensity of each cell reflects the homology between 15 candidate proteins of HBS1 (serotype 1) and those from other serotypes of *A. pleuropneumoniae*. (B) Scatter plot illustrating the homology of 15 proteins with swine proteins.

**Supplementary Figure 6**


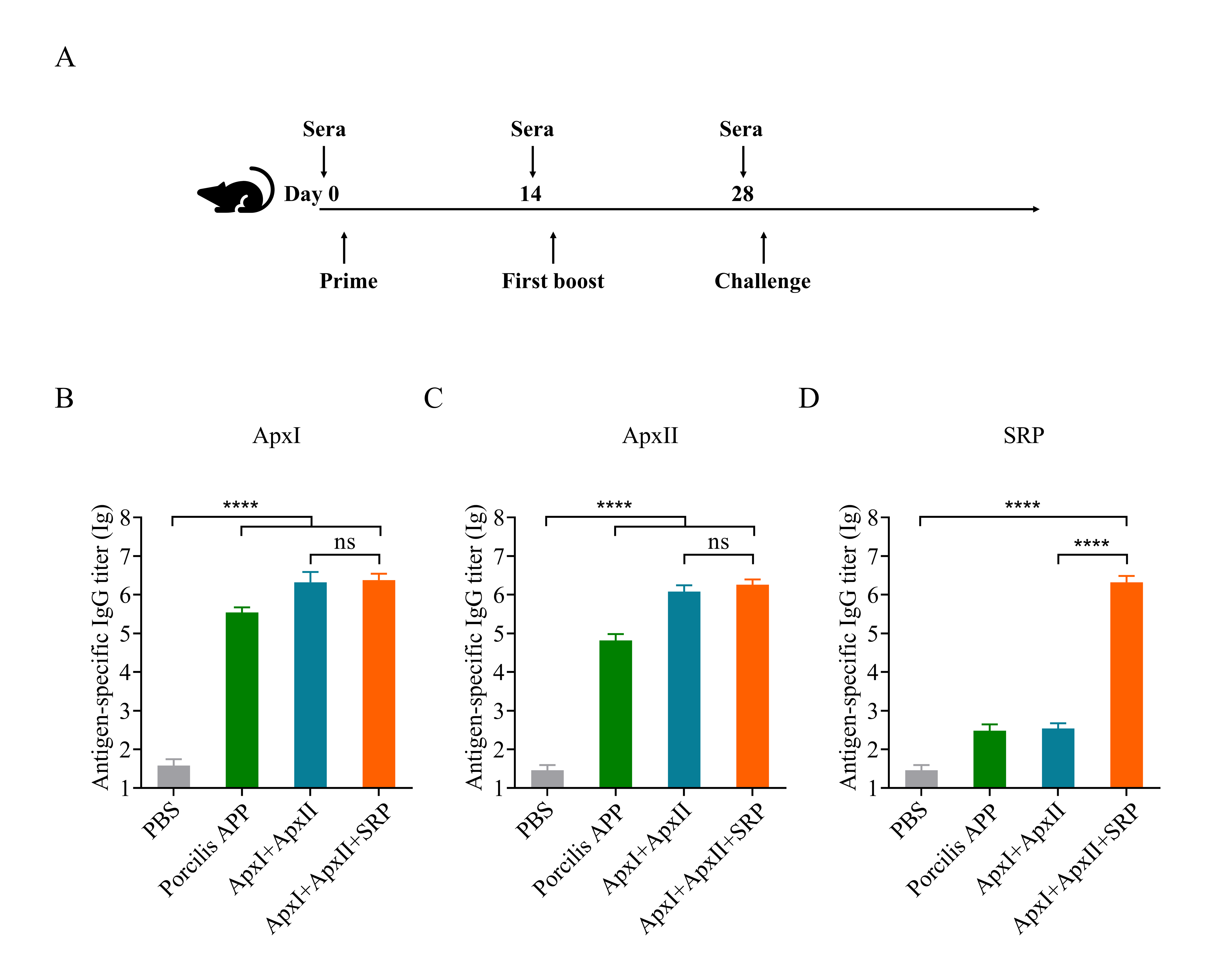


**Figure S6 Evaluation of humoral immune responses to a multivalent subunit vaccine containing HBS1_14 (SRP) and toxin proteins.** (A) Schematic representation of mouse immunization and challenge procedure. Lung tissues are collected at 12, 36, and 72 hours post-challenge for bacterial load determination and pathological analysis. The IgG endpoint titers against ApxI (B), ApxII (C), and SRP (D) were determined by ELISA. Data are presented as means ± S.D. **** indicates p < 0.0001, and ns indicates no significant difference (ANOVA).

**Supplementary Table 1**

Table S1 Determination of the median lethal dose (LD_50_) of APP HBS1 in mice

| Group | Dosage | Number | Death | Survival | Accumulation | | | |
| --- | --- | --- | --- | --- | --- | --- | --- | --- |
|  |  |  |  |  | S | D | Ratio | Mortality |
| Group 1 | 3×10^7^ | 5 | 5 | 0 | 0 | 9 | 9/9 | 100% |
| Group 2 | 1.5×10^7^ | 5 | 3 | 2 | 2 | 4 | 4/6 | 66.7% |
| Group 3 | 7.5×10^6^ | 5 | 1 | 4 | 6 | 1 | 1/7 | 14.3% |
| Group 4 | 3.75×10^6^ | 5 | 0 | 5 | 11 | 0 | 0/11 | 0 |
| Control | / | 5 | 0 | 5 | / | | | |

Note: S, survival; D, death.

**Supplementary Table 2**

Table S2 Primers for PCR and serotype identification

| Name | Sequence (5’-3’) | Length (bp) |
| --- | --- | --- |
| **APXIVANEST-1L** | GGGGACGTAACTCGGTGATT | 377 |
| APXIVANEST-1R | GCTCACCAACGTTTGCTCAT |  |
| APP-S1F | GGGCAAGCCTCTGCTCGTAA | 754 |
| APP-S1R | GAAAGAACCAAGCTCCTGCAAT |  |
| APP-S2F | ACTATGGCAATCAGTCGATTCAT | 500 |
| APP-S2R | CCTAATCGGAAACGCCATTCTG |  |
| APP-S3F | AACAAATAAAGTTGCTCGAAAGTA | 921 |
| APP-S3R | TTTGCGCTGTAGTGCTCCAAT |  |
| APP-S5F | TTTATCACTATCACCGTCCACACCT | 1100 |
| APP-S5R | CATTCGGGTCTTGTGGCTACTAA |  |
| APP-S6F | ACCACTCACTTTCCACATTAG | 720 |
| APP-S6R | AATCGGAAGGTTTTGGTCTCGTG |  |
| APP-S7F | GGTGACTGGCGTACGCCAAA | 396 |
| APP-S7R | GGGCTGCAGACTGACGTAA |  |
| APP-S8F | AACGGCTTTTGAACAACTTTATTTATTT | 977 |
| APP-S8R | TTCATTCCTAAACTCCGTATTGTCA |  |
| APP-S12F | GGTTCTCCAGATGACTCTGAAA | 559 |
| APP-S12R | GCTATTGGATGAAGATGACTCAT |  |

**Supplementary Table 3**

Table S3 Primes for PCR

| Name | Sequence（5’ to 3’） | Length (bp) |
| --- | --- | --- |
| PCR1-F | CCGAACGCAGCAAACTACGC | 1060 bp |
| PCR1-R | TTGTCTTCCTAAGACCGCTTGGCCTCCGACTT-GGGGTTAACTAGTTACTCGAGTGCGG |  |
| PCR2-F | GAACGACATGGCTACGATCCGACTT-TCGTATTCCAGTCAGGTGTGATGCTCGG | 700 bp |
| PCR2-R | TGTGAGCCAAGGAGTTG-XXXXXXXXXX-TTGTCTTCCTAAGACCGCTTGGCCT |  |

Note: The blue letter G in primer PCR2_F represents the phosphorylation of the first nucleotide at the 5' end, as required by the MGI platform. The red portion in primer PCR2_R is complementary to the barcode sequence.

**Supplementary Table 4**

Table S4 Primers for the construction of protein expression plasmid

| Name | Sequence (5’-3’) | Length (bp) |
| --- | --- | --- |
| HBS1_01F | GGAATTCCATATGCATGCGGATCATGATCAT | 529 |
| HBS1_01R | CCGCTCGAGTTTGATGACGCCGCAAG |  |
| HBS1_02F | CGCGGATCCATGAACAAAACTGAGT | 291 |
| HBS1_02R | CCGCTCGAGTTATTTTACTAAATCTTTTA |  |
| HBS1_03F | CGCGGATCCGCTTGCAGCAGCTCATC | 423 |
| HBS1_03R | CCGCTCGAGTTAGTATTCTAATACTG |  |
| HBS1_04F | CGCGGATCCCATCACGATACGAGCAATC | 768 |
| HBS1_04R | CCGCTCGAGTTTGCCGTCCCAAGCCTTA |  |
| HBS1_05F | CGCGGATCCTATCAAGCGGATAAGACGT | 1599 |
| HBS1_05R | CCGCTCGAGTTTAAAAATAACGTCTTCT |  |
| HBS1_06F | CGCGGATCCCAAGTTATTTTATTAGAT | 465 |
| HBS1_06R | CCGCTCGAGTTATTCAGCAACTACGTTTA |  |
| HBS1_07F | *TAAGAAGGAGATATACA*TATGGCGGATCAAAAATTCATTG | 687 |
| HBS1_07R | *GTGGTGGTGGTGGTGC*TCGAGTTTTTTCGCTTCGGCCG |  |
| HBS1_08F | CGCGGATCCATGTCATTAACTAACGAACA | 384 |
| HBS1_08R | CCGCTCGAGTTTGATTTCTACTTTCGCG |  |
| HBS1_09F | CGCGGATCCGCAGATCCGGTCGAGG | 582 |
| HBS1_09R | CCGCTCGAGTTACTTTTTAATTAAAAAAG |  |
| HBS1_10F | CGCGGATCCATGGAACAGTTAGAACA | 1131 |
| HBS1_10R | CCGCTCGAGGCAATCTTCATCGTTGAGTA |  |
| HBS1_11F | CGCGGATCCGAACCGACCGAAGTGCAAC | 576 |
| HBS1_11R | CCGCTCGAGAAACGCCCATTGATCAAC |  |
| HBS1_12F | CGCGGATCCATGGGCTTTTTCGACAAAT | 516 |
| HBS1_12R | CCGCTCGAGTTTGGTTAAGGTTAATAC |  |
| HBS1_13F | CGCGGATCCATGCATTTTAAACTTAATC | 1797 |
| HBS1_13R | CCGCTCGAGTTTTTCTATTTGTCGTTTTG |  |
| HBS1_14F | CGCGGATCCATGTTTGAAAACTTATCCG | 1407 |
| HBS1_14R | CCGCTCGAGGCGTTTACCGAACATATTG |  |
| HBS1_15F | CGCGGATCCGAAAATGCGCCCGCACCG | 820 |
| HBS1_15R | CCGCTCGAGCATCGAAATAATTACACA |  |
| ApxI-F | CGCGGATCCGTCAAAGGATTGATTGATT | 3051 |
| ApxI-R | CCGCTCGAGTTATAAAGAATAACTCAAAGAA |  |
| ApxII-F | CGCGGATCCATGTCAAAAATCACTTTGTCATC | 2886 |
| ApxII-R | CCGCTCGAGAGCGGCTCTAGCTAATTG |  |

Note: The underlined sequences in the table represent various enzyme cleavage sites, while the italicized sequences represent homologous sequences to the vector. The HBS1_07 expression plasmid is constructed using Gibson Assembly, whereas the remaining expression plasmids are constructed by enzyme digestion and ligation.

**Supplementary Table 5**

Table S5 Information of *A. pleuropneumoniae* immunogenic proteins

| Number | Locus_tag | Description | Name |
| --- | --- | --- | --- |
| HBS1_01 | PE794_00020 | Superoxide dismutase [Cu-Zn] SodC | SodC |
| HBS1_02 | PE794_00610 | HU family DNA-binding protein | HupA |
| HBS1_03 | PE794_01595 | Peptidoglycan-associated lipoprotein Pal | PalA |
| HBS1_04 | PE794_02090 | 5'-nucleotidase, lipoprotein e(P4) family | / |
| HBS1_05 | PE794_04100 | Bifunctional UDP-sugar hydrolase/5'-nucleotidase UshA | UshA |
| HBS1_06 | PE794_06390 | 50S ribosomal protein L9 | rplI |
| HBS1_07 | PE794_09010 | FKBP-type peptidyl-prolyl cis-trans isomerase | FkpA |
| HBS1_08 | PE794_09430 | 50S ribosomal protein L7/L12 | rplL |
| HBS1_09 | PE794_10255 | DsbA family protein | DsbA_2 |
| HBS1_10 | PE794_01470 | Spermidine/putrescine ABC transporter ATP-binding protein PotA | PotA |
| HBS1_11 | PE794_04155 | Lipoprotein insertase outer membrane protein LolB | LolB |
| HBS1_12 | PE794_07215 | PTS glucose transporter subunit IIA | crr |
| HBS1_13 | PE794_08645 | Transferrin-binding protein-like solute binding protein | TbpB |
| HBS1_14 | PE794_09570 | Signal recognition particle protein | SRP |
| HBS1_15 | PE794_10265 | Cell division protein FtsN | FtsN |

Note: “/” indicates the proteins for which the name has not been given.

**Supplementary Table 6**

Table S6 Information of 129 secreted proteins

| Locus_tag | Description | Name |
| --- | --- | --- |
| PE794_00030 | porin | / |
| PE794_00180 | deoxyribose-phosphate aldolase | / |
| PE794_00185 | glycine zipper 2TM domain-containing protein | / |
| PE794_00245 | porin family protein | / |
| PE794_00460 | YidB family protein | / |
| PE794_00645 | L,D-transpeptidase family protein | / |
| PE794_00665 | YcfL family protein | / |
| PE794_00710 | hypothetical protein | / |
| PE794_00715 | L-asparaginase 2 | / |
| PE794_00820 | FAD:protein FMN transferase | / |
| PE794_01040 | type IV pilus secretin PilQ | PilQ |
| PE794_01135 | CsgG/HfaB family protein | / |
| PE794_01140 | DUF4810 domain-containing protein | / |
| PE794_01145 | DUF799 domain-containing protein | / |
| PE794_01225 | copper resistance protein NlpE | / |
| PE794_01270 | transferrin-binding protein-like solute binding protein | / |
| PE794_01275 | lactoferrin/transferrin family TonB-dependent receptor | / |
| PE794_01355 | TolC family protein | / |
| PE794_01450 | TonB-dependent receptor | / |
| PE794_01545 | DUF5339 domain-containing protein | / |
| PE794_01590 | Tol-Pal system beta propeller repeat protein TolB | TolB |
| PE794_01595 | peptidoglycan-associated lipoprotein Pal | PalA |
| Locus_tag | Description | Name |
| PE794_01815 | lipopolysaccharide transport periplasmic protein LptA | LptA |
| PE794_01940 | NlpC/P60 family protein | / |
| PE794_01965 | S8 family serine peptidase | / |
| PE794_02090 | 5'-nucleotidase, lipoprotein e(P4) family | / |
| PE794_02205 | OmpH family outer membrane protein | / |
| PE794_02210 | outer membrane protein assembly factor BamA | BamA |
| PE794_02255 | serine hydrolase | / |
| PE794_02295 | outer membrane protein assembly factor BamE | BamE |
| PE794_02425 | galactose/glucose ABC transporter substrate-binding protein MglB | MglB |
| PE794_02475 | OmpA family protein | / |
| PE794_02545 | outer membrane lipoprotein chaperone LolA | LolA |
| PE794_02860 | phage tail tape measure protein | / |
| PE794_02905 | DUF1007 family protein | / |
| PE794_03010 | tetratricopeptide repeat protein | / |
| PE794_03040 | type II and III secretion system protein family protein | / |
| PE794_03080 | ABC transporter substrate-binding protein | / |
| PE794_03090 | TonB-dependent receptor | / |
| PE794_03095 | ABC transporter ATP-binding protein/permease | / |
| PE794_03240 | hypothetical protein | / |
| PE794_03335 | NlpC/P60 family protein | / |
| PE794_03495 | porin | / |
| PE794_03625 | hypothetical protein | / |
| PE794_03645 | transglycosylase SLT domain-containing protein | / |
| PE794_03975 | alkaline phosphatase | / |
| PE794_04155 | lipoprotein insertase outer membrane protein LolB | LolB |
| Locus_tag | Description | Name |
| PE794_04190 | single-stranded DNA-binding protein | / |
| PE794_04355 | murein transglycosylase A | mltA |
| PE794_04375 | hydroxyisourate hydrolase | uraH |
| PE794_04430 | autotransporter assembly complex protein TamA | / |
| PE794_04440 | murein hydrolase activator EnvC | EnvC |
| PE794_04485 | TolC family protein | / |
| PE794_04565 | hypothetical protein | / |
| PE794_04610 | hypothetical protein | / |
| PE794_04735 | YdgA family protein | / |
| PE794_04750 | formate dehydrogenase-N subunit alpha | fdnG |
| PE794_05085 | MetQ/NlpA family ABC transporter substrate-binding protein | / |
| PE794_05090 | MetQ/NlpA family lipoprotein | / |
| PE794_05125 | TonB-dependent receptor | / |
| PE794_05130 | hypothetical protein | / |
| PE794_05135 | surface lipoprotein assembly modifier | / |
| PE794_05190 | outer membrane protein transport protein | / |
| PE794_05275 | RTX family hemolysin | / |
| PE794_05300 | hemagglutinin repeat-containing protein | / |
| PE794_05305 | ShlB/FhaC/HecB family hemolysin secretion/activation protein | / |
| PE794_05320 | LPS assembly protein LptD | / |
| PE794_05490 | calcium-binding protein | / |
| PE794_05735 | TonB-dependent hemoglobin/transferrin/lactoferrin family receptor | / |
| PE794_05800 | NirD/YgiW/YdeI family stress tolerance protein | / |
| PE794_05815 | YtfJ family protein | / |
| PE794_05965 | outer membrane beta-barrel protein | / |
| Locus_tag | Description | Name |
| PE794_06005 | family 20 glycosylhydrolase | / |
| PE794_06040 | hypothetical protein | / |
| PE794_06130 | outer membrane protein assembly factor BamD | / |
| PE794_06205 | N-acetylmuramoyl-L-alanine amidase | / |
| PE794_06305 | hypothetical protein | / |
| PE794_06380 | hypothetical protein | / |
| PE794_06720 | maltose operon protein MalM | MalM |
| PE794_06725 | maltoporin | / |
| PE794_06910 | type IV pilus biogenesis/stability protein PilW | / |
| PE794_07010 | rhodanese-like domain-containing protein | / |
| PE794_07055 | DegQ family serine endoprotease | / |
| PE794_07075 | hypothetical protein | / |
| PE794_07085 | TonB-dependent receptor | / |
| PE794_07115 | hypothetical protein | / |
| PE794_07390 | transporter substrate-binding domain-containing protein | / |
| PE794_07450 | division/outer membrane stress-associated lipid-binding lipoprotein | / |
| PE794_07655 | hypothetical protein | / |
| PE794_07735 | hypothetical protein | / |
| PE794_07840 | SIMPL domain-containing protein | / |
| PE794_07900 | RsiV family protein | / |
| PE794_07975 | porin OmpA | OmpA |
| PE794_08090 | MliC family protein | / |
| PE794_08195 | patatin-like phospholipase family protein | / |
| PE794_08640 | lactoferrin/transferrin family TonB-dependent receptor | / |
| PE794_08645 | transferrin-binding protein-like solute binding protein | tbpB |
| Locus_tag | Description | Name |
| PE794_08695 | hypothetical protein | / |
| PE794_08720 | polysaccharide export protein | / |
| PE794_08750 | TAXI family TRAP transporter solute-binding subunit | / |
| PE794_08790 | serine hydrolase | / |
| PE794_08795 | septal ring lytic transglycosylase RlpA family protein | / |
| PE794_09010 | FKBP-type peptidyl-prolyl cis-trans isomerase | FkpA |
| PE794_09305 | hypothetical protein | / |
| PE794_09370 | porin family protein | / |
| PE794_09545 | membrane-bound lytic murein transglycosylase MltC | MltC |
| PE794_09590 | transferrin-binding protein-like solute binding protein | / |
| PE794_09925 | MipA/OmpV family protein | / |
| PE794_10055 | hypothetical protein | / |
| PE794_10180 | porin OmpA | OmpA |
| PE794_10290 | DUF5358 family protein | / |
| PE794_10305 | TonB-dependent receptor plug domain-containing protein | / |
| PE794_10325 | pitrilysin | ptrA |
| PE794_10400 | M48 family metallopeptidase | / |
| PE794_10480 | M13 family metallopeptidase | / |
| PE794_10505 | VacJ family lipoprotein | / |
| PE794_10520 | poly-beta-1,6 N-acetyl-D-glucosamine export porin PgaA | PgaA |
| PE794_10525 | poly-beta-1,6-N-acetyl-D-glucosamine N-deacetylase PgaB | PgaB |
| PE794_10560 | LysM peptidoglycan-binding domain-containing protein | / |
| PE794_10565 | hypothetical protein | / |
| PE794_10570 | murein hydrolase activator NlpD | NlpD |
| PE794_10650 | ricin-type beta-trefoil lectin domain protein | / |
| Locus_tag | Description | Name |
| PE794_10675 | iron transporter | / |
| PE794_10680 | TonB-dependent receptor | / |
| PE794_10685 | transferrin-binding protein-like solute binding protein | / |
| PE794_10925 | surface lipoprotein assembly modifier | / |
| PE794_10690 | transferrin-binding protein-like solute binding protein | / |
| PE794_10995 | TonB-dependent siderophore receptor | / |
| PE794_11130 | glycoside hydrolase family 43 C-terminal domain-containing protein | / |

Note: “/” indicates the proteins for which the name has not been given.

**Supplementary Table 7**

Table S7 Information of 333 surface proteins

| Locus_tag | Description | Name |
| --- | --- | --- |
| PE794_00020 | superoxide dismutase [Cu-Zn] SodC | SodC |
| PE794_00025 | aspartate-semialdehyde dehydrogenase | asd |
| PE794_00065 | UDP-N-acetylmuramoyl-L-alanyl-D-glutamate--2,6-diaminopimelate ligase | murE |
| PE794_00070 | UDP-N-acetylmuramoyl-tripeptide--D-alanyl-D-alanine ligase | murF |
| PE794_00080 | UDP-N-acetylmuramoyl-L-alanine--D-glutamate ligase | murD |
| PE794_00100 | D-alanine--D-alanine ligase | / |
| PE794_00110 | cell division protein FtsA | FtsA |
| PE794_00150 | peptide chain release factor 3 | prfC |
| PE794_00165 | aminoacyl-tRNA hydrolase | pth |
| PE794_00170 | redox-regulated ATPase YchF | YchF |
| PE794_00195 | arginine--tRNA ligase | / |
| PE794_00220 | isoleucine--tRNA ligase | / |
| PE794_00270 | translational GTPase TypA | TypA |
| PE794_00275 | GrxA family glutaredoxin | / |
| PE794_00290 | folate-binding protein YgfZ | / |
| PE794_00310 | tRNA (uridine(34)/cytosine(34)/5-carboxymethylaminomethyluridine(34)-2'-O)-methyltransferase TrmL | TrmL |
| PE794_00315 | transaldolase | tal |
| PE794_00345 | ABC transporter substrate-binding protein | / |
| PE794_00395 | Grx4 family monothiol glutaredoxin | GrxD |
| PE794_00530 | ammonia-forming nitrite reductase cytochrome c552 subunit | nrfA |
| PE794_00600 | uroporphyrinogen decarboxylase | hemE |
| PE794_00610 | HU family DNA-binding protein | HupA |
| Locus_tag | Description | Name |
| PE794_00615 | thymidylate synthase | / |
| PE794_00620 | hypothetical protein | / |
| PE794_00625 | biotin synthase BioB | / |
| PE794_00635 | RNA chaperone ProQ | ProQ |
| PE794_00640 | carboxy terminal-processing peptidase | prc |
| PE794_00645 | L,D-transpeptidase family protein | / |
| PE794_00670 | purine nucleoside phosphoramidase | hinT |
| PE794_00710 | hypothetical protein | / |
| PE794_00775 | ribonucleotide-diphosphate reductase subunit beta | / |
| PE794_00785 | Fe-S biogenesis protein NfuA | NfuA |
| PE794_00815 | NADH:ubiquinone reductase (Na(+)-transporting) subunit F | nqrF |
| PE794_00830 | tRNA 2-thiouridine(34) synthase MnmA | MnmA |
| PE794_00945 | lactoylglutathione lyase | gloA |
| PE794_00975 | pyruvate kinase | pyk |
| PE794_01045 | transcription antitermination factor NusB | NusB |
| PE794_01065 | TrmH family RNA methyltransferase | / |
| PE794_01070 | inositol-1-monophosphatase | suhB |
| PE794_01085 | serine hydroxymethyltransferase | / |
| PE794_01160 | 50S ribosomal protein L20 | rplT |
| PE794_01180 | hemolysin family protein | / |
| PE794_01210 | YajG family lipoprotein | / |
| PE794_01240 | preprotein translocase subunit SecA | SecA |
| PE794_01255 | N-acetyl-gamma-glutamyl-phosphate reductase | argC |
| PE794_01265 | aspartate aminotransferase family protein | / |
| PE794_01280 | transcription termination factor Rho | Rho |
| Locus_tag | Description | Name |
| PE794_01285 | cysteine synthase A | cysK |
| PE794_01300 | superoxide dismutase [Mn] | sodA |
| PE794_01340 | aminoacyl-histidine dipeptidase | / |
| PE794_01350 | guanylate kinase | gmk |
| PE794_01355 | TolC family protein | / |
| PE794_01380 | molybdate ABC transporter substrate-binding protein | modA |
| PE794_01400 | adenine phosphoribosyltransferase | apt |
| PE794_01430 | metal ABC transporter substrate-binding protein | / |
| PE794_01470 | spermidine/putrescine ABC transporter ATP-binding protein PotA | PotA |
| PE794_01500 | DNA topoisomerase (ATP-hydrolyzing) subunit A | gyrA |
| PE794_01535 | ABC transporter ATP-binding protein | / |
| PE794_01585 | cell envelope integrity protein TolA | TolA |
| PE794_01590 | Tol-Pal system beta propeller repeat protein TolB | TolB |
| PE794_01685 | 5-methyltetrahydropteroyltriglutamate--homocysteine S-methyltransferase | / |
| PE794_01855 | 1-phosphofructokinase | fruK |
| PE794_01860 | fused PTS fructose transporter subunit IIA/HPr protein | fruB |
| PE794_01900 | methionine--tRNA ligase | metG |
| PE794_01950 | autonomous glycyl radical cofactor GrcA | GrcA |
| PE794_01985 | extracellular solute-binding protein | / |
| PE794_02010 | DUF2057 domain-containing protein | / |
| PE794_02020 | glycerol kinase GlpK | GlpK |
| PE794_02025 | endopeptidase La | lon |
| PE794_02045 | glycerol-3-phosphate dehydrogenase subunit GlpB | GlpB |
| PE794_02065 | bifunctional 3,4-dihydroxy-2-butanone-4-phosphate synthase/GTP cyclohydrolase II | / |
| PE794_02080 | KpsF/GutQ family sugar-phosphate isomerase | / |
| Locus_tag | Description | Name |
| PE794_02090 | 5'-nucleotidase, lipoprotein e(P4) family | / |
| PE794_02105 | Cof-type HAD-IIB family hydrolase | / |
| PE794_02135 | uracil phosphoribosyltransferase | upp |
| PE794_02140 | 16S rRNA (adenine(1518)-N(6)/adenine(1519)-N(6))-dimethyltransferase RsmA | RsmA |
| PE794_02145 | peptidylprolyl isomerase | / |
| PE794_02185 | 1-deoxy-D-xylulose-5-phosphate reductoisomerase | ispC |
| PE794_02235 | N-acetylglucosamine kinase | nagK |
| PE794_02300 | nucleoid-associated protein YejK | YejK |
| PE794_02330 | peptide-methionine (R)-S-oxide reductase MsrB | MsrB |
| PE794_02335 | type I glyceraldehyde-3-phosphate dehydrogenase | gap |
| PE794_02395 | lactate utilization protein C | / |
| PE794_02405 | (Fe-S)-binding protein | / |
| PE794_02430 | succinate--CoA ligase subunit alpha | sucD |
| PE794_02435 | ADP-forming succinate--CoA ligase subunit beta | sucC |
| PE794_02445 | 2-oxoglutarate dehydrogenase complex dihydrolipoyllysine-residue succinyltransferase | odhB |
| PE794_02465 | bifunctional protein-disulfide isomerase/oxidoreductase DsbC | DsbC |
| PE794_02475 | OmpA family protein | / |
| PE794_02480 | pyridoxal phosphatase | / |
| PE794_02545 | outer membrane lipoprotein chaperone LolA | LolA |
| PE794_02610 | malic enzyme | / |
| PE794_02905 | DUF1007 family protein | / |
| PE794_02970 | translation elongation factor 4 | lepA |
| PE794_02980 | ribonuclease III | rnc |
| PE794_03065 | ATP-dependent RNA helicase RhlB | RhlB |
| PE794_03075 | DUF853 family protein | / |
| Locus_tag | Description | Name |
| PE794_03080 | ABC transporter substrate-binding protein | / |
| PE794_03105 | 30S ribosomal protein S2 | rpsB |
| PE794_03110 | translation elongation factor Ts | tsf |
| PE794_03125 | ribosome recycling factor | frr |
| PE794_03150 | DEAD/DEAH box helicase | / |
| PE794_03230 | phospho-sugar mutase | / |
| PE794_03315 | NAD(P)H-dependent oxidoreductase | / |
| PE794_03360 | leucine-responsive transcriptional regulator Lrp | Lrp |
| PE794_03365 | DNA translocase FtsK 4TM domain-containing protein | / |
| PE794_03425 | acetyl-CoA carboxylase, carboxyltransferase subunit beta | accD |
| PE794_03440 | ribosome maturation factor RimP | RimP |
| PE794_03445 | transcription termination factor NusA | NusA |
| PE794_03450 | translation initiation factor IF-2 | infB |
| PE794_03470 | DUF1508 domain-containing protein | / |
| PE794_03475 | phosphate acetyltransferase | pta |
| PE794_03480 | acetate kinase | / |
| PE794_03485 | 2',3'-cyclic-nucleotide 2'-phosphodiesterase | cpdB |
| PE794_03510 | phosphomannomutase/phosphoglucomutase | / |
| PE794_03520 | alanine--tRNA ligase | alaS |
| PE794_03535 | ClpXP protease specificity-enhancing factor | / |
| PE794_03540 | stringent starvation protein A | sspA |
| PE794_03580 | EfeM/EfeO family lipoprotein | / |
| PE794_03615 | asparagine--tRNA ligase | asnS |
| PE794_03630 | elongation factor P | efp |
| PE794_03635 | glutamine--tRNA ligase | glnS |
| Locus_tag | Description | Name |
| PE794_03665 | 23S rRNA (cytidine(2498)-2'-O)-methyltransferase RlmM | RlmM |
| PE794_03680 | trimethylamine-N-oxide reductase TorA | TorA |
| PE794_03720 | type I DNA topoisomerase | topA |
| PE794_03745 | O-acetylhomoserine aminocarboxypropyltransferase/cysteine synthase | / |
| PE794_03750 | 3-phosphoserine/phosphohydroxythreonine transaminase | serC |
| PE794_03790 | lysine-sensitive aspartokinase 3 | lysC |
| PE794_03810 | siderophore ABC transporter substrate-binding protein | / |
| PE794_03885 | acetolactate synthase small subunit | ilvN |
| PE794_03925 | orotidine-5'-phosphate decarboxylase | pyrF |
| PE794_04005 | replication initiation negative regulator SeqA | SeqA |
| PE794_04020 | SDR family oxidoreductase | / |
| PE794_04050 | bis(5'-nucleosyl)-tetraphosphatase (symmetrical) ApaH | ApaH |
| PE794_04100 | bifunctional UDP-sugar hydrolase/5'-nucleotidase UshA | UshA |
| PE794_04135 | pyruvate dehydrogenase (acetyl-transferring), homodimeric type | aceE |
| PE794_04150 | 4-(cytidine 5'-diphospho)-2-C-methyl-D-erythritol kinase | ispE |
| PE794_04190 | single-stranded DNA-binding protein | / |
| PE794_04265 | D-sedoheptulose 7-phosphate isomerase | lpcA |
| PE794_04270 | phosphoenolpyruvate carboxykinase (ATP) | pckA |
| PE794_04300 | MOSC domain-containing protein | / |
| PE794_04320 | lysine--tRNA ligase | lysS |
| PE794_04380 | HTH-type transcriptional repressor PurR | PurR |
| PE794_04390 | DNA topoisomerase (ATP-hydrolyzing) subunit B | gyrB |
| PE794_04445 | 2,3-diphosphoglycerate-dependent phosphoglycerate mutase | / |
| PE794_04525 | ABC transporter substrate-binding protein | / |
| PE794_04550 | 5-methyltetrahydropteroyltriglutamate--homocysteine S-methyltransferase | / |
| Locus_tag | Description | Name |
| PE794_04610 | hypothetical protein | / |
| PE794_04615 | CYTH domain-containing protein | / |
| PE794_04620 | YcjX family protein | / |
| PE794_04645 | dipeptidase PepE | PepE |
| PE794_04765 | formate dehydrogenase accessory protein FdhE | FdhE |
| PE794_04790 | 4-hydroxy-tetrahydrodipicolinate synthase | dapA |
| PE794_04905 | helix-turn-helix domain-containing protein | / |
| PE794_04920 | hypothetical protein | / |
| PE794_05105 | cysteine--tRNA ligase | cysS |
| PE794_05110 | peptidylprolyl isomerase | / |
| PE794_05150 | Fe-S protein assembly chaperone HscA | HscA |
| PE794_05175 | Fe-S cluster assembly scaffold IscU | IscU |
| PE794_05180 | IscS subfamily cysteine desulfurase | / |
| PE794_05230 | 8-amino-7-oxononanoate synthase | / |
| PE794_05245 | transcription elongation factor GreA | GreA |
| PE794_05275 | RTX family hemolysin | / |
| PE794_05430 | transketolase | tkt |
| PE794_05435 | molecular chaperone HtpG | / |
| PE794_05470 | galactose-1-phosphate uridylyltransferase | galT |
| PE794_05500 | iron-sulfur cluster carrier protein ApbC | ApbC |
| PE794_05535 | heme biosynthesis protein HemY | / |
| PE794_05565 | co-chaperone GroES | / |
| PE794_05575 | deoxyribose-phosphate aldolase | deoC |
| PE794_05670 | oligopeptidase A | prlC |
| PE794_05680 | formate C-acetyltransferase | pflB |
| Locus_tag | Description | Name |
| PE794_05740 | heme utilization protein HutZ | HutZ |
| PE794_05775 | beta-ketoacyl-ACP synthase I | fabB |
| PE794_05815 | YtfJ family protein | / |
| PE794_05855 | ATP-binding protein | / |
| PE794_05910 | adenylosuccinate synthase | purA |
| PE794_05935 | methionine adenosyltransferase | metK |
| PE794_05965 | outer membrane beta-barrel protein | / |
| PE794_05970 | ATP-dependent chaperone ClpB | ClpB |
| PE794_06085 | beta-N-acetylhexosaminidase | nagZ |
| PE794_06095 | phosphopyruvate hydratase | eno |
| PE794_06100 | NUDIX hydrolase | / |
| PE794_06170 | cytochrome b562 | / |
| PE794_06175 | tRNA 5-hydroxyuridine modification protein YegQ | YegQ |
| PE794_06205 | N-acetylmuramoyl-L-alanine amidase | / |
| PE794_06210 | glucose-6-phosphate isomerase | pgi |
| PE794_06235 | shikimate dehydrogenase | aroE |
| PE794_06285 | DUF302 domain-containing protein | / |
| PE794_06315 | pitrilysin family protein | / |
| PE794_06340 | 23S rRNA pseudouridine(2605) synthase RluB | RluB |
| PE794_06355 | Dyp-type peroxidase | / |
| PE794_06390 | 50S ribosomal protein L9 | rplI |
| PE794_06405 | 30S ribosomal protein S6 | rpsF |
| PE794_06555 | NAD(P)H-dependent oxidoreductase | / |
| PE794_06600 | GNAT family protein | / |
| PE794_06615 | hexitol phosphatase HxpB | HxpB |
| Locus_tag | Description | Name |
| PE794_06635 | S-ribosylhomocysteine lyase | luxS |
| PE794_06645 | ferric iron uptake transcriptional regulator | fur |
| PE794_06660 | Cys-tRNA(Pro)/Cys-tRNA(Cys) deacylase YbaK | YbaK |
| PE794_06700 | YajQ family cyclic di-GMP-binding protein | / |
| PE794_06800 | phosphoglycerate kinase | / |
| PE794_06820 | DNA topoisomerase IV subunit B | parE |
| PE794_06915 | bifunctional tRNA (adenosine(37)-C2)-methyltransferase TrmG/ribosomal RNA large subunit methyltransferase RlmN | / |
| PE794_06940 | glutamate--tRNA ligase | gltX |
| PE794_07000 | energy-dependent translational throttle protein EttA | EttA |
| PE794_07020 | UDP-N-acetylglucosamine 1-carboxyvinyltransferase | murA |
| PE794_07070 | malate dehydrogenase | mdh |
| PE794_07080 | adenylate kinase | adk |
| PE794_07095 | UDP-glucose 4-epimerase GalE | GalE |
| PE794_07105 | cell division protein ZipA | ZipA |
| PE794_07120 | decarboxylating NADP(+)-dependent phosphogluconate dehydrogenase | gnd |
| PE794_07125 | hypothetical protein | / |
| PE794_07145 | 6-phosphogluconolactonase | pgl |
| PE794_07205 | phosphocarrier protein Hpr | ptsH |
| PE794_07210 | phosphoenolpyruvate-protein phosphotransferase PtsI | PtsI |
| PE794_07215 | PTS glucose transporter subunit IIA | crr |
| PE794_07220 | tRNA (N6-isopentenyl adenosine(37)-C2)-methylthiotransferase MiaB | MiaB |
| PE794_07230 | hydrogenase nickel incorporation protein HypB | HypB |
| PE794_07325 | aminopeptidase N | pepN |
| PE794_07340 | cytidine deaminase | cdd |
| PE794_07355 | signal recognition particle-docking protein FtsY | FtsY |
| Locus_tag | Description | Name |
| PE794_07390 | transporter substrate-binding domain-containing protein | / |
| PE794_07400 | YbhB/YbcL family Raf kinase inhibitor-like protein | / |
| PE794_07435 | penicillin-binding protein activator | / |
| PE794_07450 | division/outer membrane stress-associated lipid-binding lipoprotein | dolP |
| PE794_07590 | ketoacyl-ACP synthase III | / |
| PE794_07630 | PTS mannose transporter subunit IIAB | manX |
| PE794_07650 | mannose-6-phosphate isomerase, class I | manA |
| PE794_07655 | hypothetical protein | / |
| PE794_07680 | ABC transporter substrate-binding protein | / |
| PE794_07700 | class 1 fructose-bisphosphatase | fbp |
| PE794_07710 | glutathione peroxidase | / |
| PE794_07740 | tyrosine--tRNA ligase | tyrS |
| PE794_07750 | DNA primase | dnaG |
| PE794_07755 | 30S ribosomal protein S21 | rpsU |
| PE794_07805 | dTDP-4-dehydrorhamnose 3,5-epimerase | rfbC |
| PE794_07810 | dTDP-4-dehydrorhamnose reductase | rfbD |
| PE794_07820 | dTDP-glucose 4,6-dehydratase | rffG |
| PE794_07855 | peptidylprolyl isomerase | slyD |
| PE794_07865 | phosphoglucosamine mutase | glmM |
| PE794_07870 | ribose-5-phosphate isomerase RpiA | RpiA |
| PE794_07885 | zinc ABC transporter substrate-binding protein ZnuA | ZnuA |
| PE794_07920 | NAD(P)H-dependent oxidoreductase | / |
| PE794_07935 | nitrate reductase catalytic subunit NapA | NapA |
| PE794_07980 | galactose/glucose ABC transporter substrate-binding protein MglB | MglB |
| PE794_08070 | DEAD/DEAH box helicase family protein | / |
| Locus_tag | Description | Name |
| PE794_08085 | LysM-like peptidoglycan-binding domain-containing protein | / |
| PE794_08110 | 30S ribosomal protein S7 | rpsG |
| PE794_08150 | multicopper oxidase domain-containing protein | / |
| PE794_08160 | thiol peroxidase | tpx |
| PE794_08210 | threonine synthase | thrC |
| PE794_08250 | trigger factor | tig |
| PE794_08260 | protein-export chaperone SecB | SecB |
| PE794_08300 | type I pantothenate kinase | coaA |
| PE794_08345 | NAD(P)H-binding protein | / |
| PE794_08350 | heme anaerobic degradation radical SAM methyltransferase ChuW/HutW | HutW |
| PE794_08355 | glutathione S-transferase N-terminal domain-containing protein | / |
| PE794_08385 | fumarate reductase (quinol) flavoprotein subunit | frdA |
| PE794_08420 | N-acetylmuramic acid 6-phosphate etherase | murQ |
| PE794_08425 | 50S ribosomal protein L11 methyltransferase | prmA |
| PE794_08530 | hydroxylamine reductase | hcp |
| PE794_08535 | NADH oxidoreductase | hcr |
| PE794_08750 | TAXI family TRAP transporter solute-binding subunit | / |
| PE794_08820 | DNA mismatch repair protein MutS | MutS |
| PE794_08850 | YfcZ/YiiS family protein | / |
| PE794_08930 | DUF4198 domain-containing protein | / |
| PE794_08960 | PTS mannitol transporter subunit IICBA | / |
| PE794_08965 | glutamine--fructose-6-phosphate transaminase (isomerizing) | glmS |
| PE794_08995 | 5'-methylthioadenosine/S-adenosylhomocysteine nucleosidase | mtnN |
| PE794_09010 | FKBP-type peptidyl-prolyl cis-trans isomerase | FkpA |
| PE794_09180 | ribose ABC transporter substrate-binding protein RbsB | RbsB |
| Locus_tag | Description | Name |
| PE794_09345 | FKBP-type peptidyl-prolyl cis-trans isomerase | / |
| PE794_09430 | 50S ribosomal protein L7/L12 | rplL |
| PE794_09540 | oxidative damage protection protein | / |
| PE794_09570 | signal recognition particle protein | SRP |
| PE794_09575 | bifunctional UDP-sugar hydrolase/5'-nucleotidase | / |
| PE794_09655 | 50S ribosomal protein L4 | rplD |
| PE794_09665 | 50S ribosomal protein L2 | rplB |
| PE794_09670 | 30S ribosomal protein S19 | rpsS |
| PE794_09695 | 30S ribosomal protein S17 | rpsQ |
| PE794_09700 | 50S ribosomal protein L14 | rplN |
| PE794_09715 | 30S ribosomal protein S14 | rpsN |
| PE794_09720 | 30S ribosomal protein S8 | rpsH |
| PE794_09725 | 50S ribosomal protein L6 | rplF |
| PE794_09735 | 30S ribosomal protein S5 | rpsE |
| PE794_09765 | 30S ribosomal protein S11 | rpsK |
| PE794_09770 | 30S ribosomal protein S4 | rpsD |
| PE794_09790 | ribosome maturation factor RimM | RimM |
| PE794_09795 | tRNA (guanosine(37)-N1)-methyltransferase TrmD | TrmD |
| PE794_09800 | 50S ribosomal protein L19 | rplS |
| PE794_09810 | ABC transporter substrate-binding protein | / |
| PE794_09830 | ABC transporter substrate-binding protein | / |
| PE794_09855 | glycine--tRNA ligase subunit alpha | glyQ |
| PE794_09870 | glycine--tRNA ligase subunit beta | glyS |
| PE794_09960 | acyl carrier protein | acpP |
| PE794_10030 | protein-methionine-sulfoxide reductase catalytic subunit MsrP | MsrP |
| Locus_tag | Description | Name |
| PE794_10065 | aspartate--ammonia ligase | asnA |
| PE794_10110 | assimilatory sulfite reductase (NADPH) hemoprotein subunit | cysI |
| PE794_10120 | GTP-binding protein | / |
| PE794_10125 | sulfate adenylyltransferase subunit CysD | CysD |
| PE794_10140 | sulfate ABC transporter substrate-binding protein | / |
| PE794_10195 | transglutaminase family protein | / |
| PE794_10220 | 2,3,4,5-tetrahydropyridine-2,6-dicarboxylate N-succinyltransferase | dapD |
| PE794_10240 | acetyl-CoA carboxylase biotin carboxylase subunit | accC |
| PE794_10255 | DsbA family protein | DsbA_2 |
| PE794_10265 | cell division protein FtsN | FtsN |
| PE794_10315 | ATP-grasp domain-containing protein | / |
| PE794_10320 | alpha/beta hydrolase-fold protein | / |
| PE794_10325 | pitrilysin | ptrA |
| PE794_10355 | bifunctional 3-hydroxydecanoyl-ACP dehydratase/trans-2-decenoyl-ACP isomerase | fabA |
| PE794_10400 | M48 family metallopeptidase | / |
| PE794_10405 | inorganic diphosphatase | ppa |
| PE794_10445 | molecular chaperone DnaK | DnaK |
| PE794_10540 | triose-phosphate isomerase | tpiA |
| PE794_10545 | tRNA pseudouridine(13) synthase TruD | TruD |
| PE794_10560 | LysM peptidoglycan-binding domain-containing protein | / |
| PE794_10570 | murein hydrolase activator NlpD | NlpD |
| PE794_10700 | alcohol dehydrogenase AdhP | AdhP |
| PE794_10730 | cAMP-activated global transcriptional regulator CRP | CRP |
| PE794_10745 | dUTP diphosphatase | dut |
| PE794_10775 | hypothetical protein | / |
| Locus_tag | Description | Name |
| PE794_10875 | 3-oxoacyl-ACP reductase FabG | FabG |
| PE794_10880 | ACP S-malonyltransferase | fabD |
| PE794_10900 | NAD(P)H-dependent oxidoreductase | rplU |
| PE794_10910 | 50S ribosomal protein L21 | / |
| PE794_10930 | Cof-type HAD-IIB family hydrolase | / |
| PE794_11065 | type I methionyl aminopeptidase | map |
| PE794_11070 | thioredoxin family protein | / |
| PE794_11080 | glycoside hydrolase family 32 protein | / |
| PE794_11085 | aminoimidazole riboside kinase | / |
| PE794_11115 | 3-deoxy-8-phosphooctulonate synthase | kdsA |
| PE794_11135 | peptide chain release factor 1 | prfA |

Note: “/” indicates the proteins for which the name has not been given.
